# Impaired adipogenesis and lipid storage capacity in subcutaneous adipose tissue of patients with PMOS

**DOI:** 10.1172/jci.insight.199981

**Published:** 2026-08-10

**Authors:** Adeline Divoux, Edina Erdos, Katie L. Whytock, Timothy F. Osborne, Steven R. Smith

**Affiliations:** 1 Translational Research Institute, AdventHealth, Orlando, Florida, USA.; 2 Division of Diabetes Endocrinology and Metabolism, Departments of Medicine, Pediatrics, and Physiology Pharmacology, and Therapeutics, Johns Hopkins University School of Medicine, and; 3 Institute for Fundamental Biomedical Research, Johns Hopkins All Children’s Hospital, St. Petersburg, Florida, USA.; 4 Institute of Genetics, HUN-REN Biological Research Centre, Szeged, Hungary.

**Keywords:** Cell biology, Metabolism, Adipose tissue, Extracellular matrix, Human stem cells

## Abstract

Women with PMOS (formally termed PCOS) have an overall increased prevalence of metabolic syndrome (MetS) and central obesity. To help determine whether there might be changes in s.c. adipose tissue (SAT) associated with these abnormalities, we performed single-nuclei and scRNA-seq on SAT biopsies from 15 premenopausal PMOS women with signs of insulin resistance and 17 healthy BMI-matched controls. In SAT from PMOS versus control we observed a higher ratio of fibrotic versus insulin sensitive adipocytes and a higher ratio of mesenchymal stem cells (MSCs) to preadipocytes. Further in silico analysis suggested that preadipocytes in PMOS are more inflammatory and have a reduced capacity for differentiation. Slit homolog 2 (SLIT2), which is expressed at higher levels in MSC from PMOS, decreased adipogenesis in cell culture assays likely through its interaction with the Roundabout homolog 1 and homolog 2 (ROBO1/2) receptor expressed on the surface of preadipocytes. These new observations are consistent with higher SLIT/ROBO signaling, leading to reduced differentiation in the SAT of PMOS as an underlying mechanism for the aberrant ectopic fat accumulation and the development of MetS in PMOS.

## Abstract 2

Women with PCOS show altered fat tissue specifically poor cell maturation, driven partly by increased SLIT/ROBO signaling, contributing to metabolic syndrome risk.

## Graphical abstract

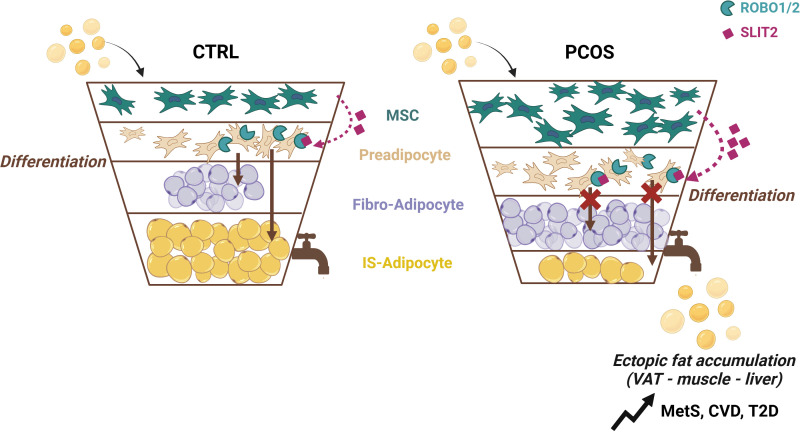

## Introduction

Polyendocrine metabolic ovarian syndrome (PMOS) is the most common hormonal disturbance in women and occurs in up to 18% of females of reproductive age (1). PMOS is a complex disease characterized by ovulatory dysfunction and excess circulating androgens. Importantly, insulin resistance (IR) in target tissues (muscle, adipose tissue [AT], liver) is found in 44%–85% of the women with PMOS (2, 3). PMOS increases the risk for type 2 diabetes mellitus (T2D) and metabolic syndrome, both of which are strong risk factors for cardiovascular disease (CVD) (4, 5). In addition, IR is known to lower circulating sex hormone-binding globulin (SHBG), which also leads to an increase in free androgens (6). Elevated androgens constrain normal follicle formation, resulting in irregular menses. Understanding the etiology of IR in PMOS could help develop better strategies to prevent and treat these metabolic diseases.

Patients with PMOS have increased visceral adipose tissue (VAT) mass, which is correlated with androgen levels and the degree of IR (7). However, analysis of computerized tomography (CT) scans have revealed that, in addition to VAT mass, s.c. adipose tissue (SAT) mass was independently and positively correlated with insulin sensitivity as measured by a euglycemic hyperinsulinemic clamp (8). Some studies suggest that alteration of SAT function is a key determinant of IR and metabolic abnormalities in PMOS (9). These studies show that, in women with PMOS, the SAT includes high level of hypertrophic adipocytes (10, 11), which secrete more inflammatory cytokines (e.g., TNF-α) and free fatty acids (FFAs), leading to the impairment in both lipolysis and insulin action (12, 13) and less adiponectin (14). Importantly, SAT dysfunction has been observed even in lean PMOS individuals (15). The stroma vascular fraction (SVF) of adipose tissue contains many other cell types besides adipocytes. These include stem cells, immune cells, and vascular cells, which together define the phenotype of the tissue. Their implication in PMOS pathology has never been thoroughly studied.

SAT is also distributed throughout the body and can largely be separated into 2 major compartments according to anatomical location (upper and lower body). Expansion of upper body SAT as well as expansion of abdominal SAT are referred to as abdominal (ABD) in this paper; these expansions results in an android body type body fat distribution and an increased risk of developing IR, diabetes, and CVD relative to females matched for BMI but who carry their excess weight in gluteal/thigh adipose tissue (gynoid) (16–18). In contrast, expansion of lower body SAT (referred as gluteofemoral or GF in this paper) is associated with healthier measures of cardiometabolic health (16–18).

Elliot Danforth proposed the hypothesis that, once the SAT expansion limit is reached, adipose tissue ceases to store energy efficiently and lipids begin to spill over and accumulate in other tissues (19). The latter is called ectopic fat (20, 21). This hypothesis is the foundation of the theory that ectopic lipid accumulation in nonadipocyte cells causes organic specific lipotoxic effects including IR, apoptosis, and inflammation (22). Overall, this hypothesis suggests that a failure of adipocyte differentiation occurs upstream of the ectopic fat accumulation and IR and is observed in some but not all patients with obesity, depending on the amount and the location of the adipose tissue. Furthermore, this model predicts that if there is a block in preadipocyte differentiation, precursor number would increase to maintain the lipid storage capacity of the SAT. To test the Danforth hypothesis in PMOS, we applied deep clinical phenotyping and single-cell/single-nuclei RNA-seq on SAT biopsies. Together, these methodologies allow for the determination and comparison of the cellular composition and transcriptional landscape of human SAT between PMOS and controls.

Using these advanced techniques, we provide the first evidence to our knowledge that the proportion of adipocyte precursors is altered in PMOS. This finding is consistent with a defect in adipocyte differentiation leading to ectopic fat deposition, IR, and an increased risk for cardiometabolic disease in PMOS.

## Results

### Patients with PMOS show signs of IR that correlate with adipocyte size.

**Figure 1:**
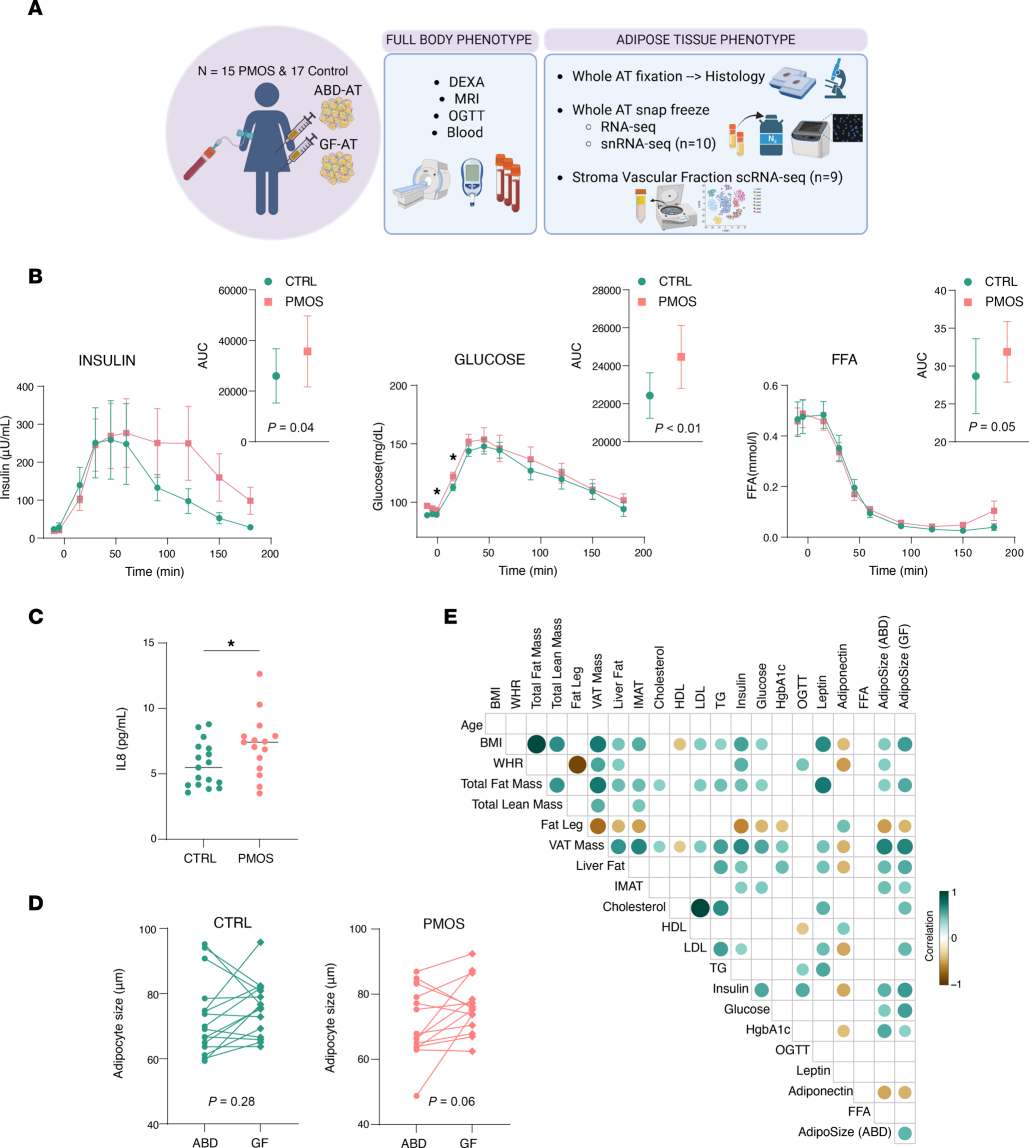
General design of the translational study and clinical characteristics of the 15 CTRL and 17 PMOS women. (**A**) General design of clinical and adipose tissue phenotyping studies. ABD-AT, s.c. abdominal adipose tissue; GF-AT, gluteofemoral adipose tissue; snRNA-seq, single-nuclei RNA-seq. (**B**) Insulin; glucose and free fatty acids (FFA) were quantified in serum samples collected during an OGTT test. *n* = 15 PMOS and 17 CTRL. *P < 0.05 Wilcoxon test between group at specific time point. AUC for each group have been reported as total area under the curve, plotted and compared using an unpaired 2-tailed *t* test. (**C**) IL-8 was measured in the serum samples of 15 PMOS and 17 CTRL women. *P < 0.05 unpaired 2-tailed *t* test. (**D**) Adipocyte size was determined by microscopy. Average of 100 adipocytes per sample is represented in ABD and GF depot. CTRL group samples are represented in green, and PMOS group samples are represented in pink. *P < 0.05 paired 2-tailed *t* test. (**E**) Correlation matrix of relevant clinical parameters and ABD and GF-AT adipocyte size. *n* = 15 PMOS and 17 CTRL. Only significant correlations (*P* < 0.05) are represented by a dot; negative correlations are depicted in brown and positive correlations are depicted in green. The size of the dot indicates the R coefficient.

We recruited 15 women with PMOS and 17 BMI-matched healthy females (CTRL). Figure 1A summarizes the methods used to measure adipose tissue distribution (dual x-ray absorptiometry [DEXA], MRI), glucose tolerance (oral glucose-tolerance test [OGTT]), IR, systemic inflammation, and dyslipidemia. In addition, we deeply interrogated biopsies from ABD and GF-SAT (histology, RNA-seq, single-nuclei RNA-seq and scRNA-seq).

The characteristics of the study groups are presented in Table 1. Although the women with PMOS were slightly younger than the CTRL individuals, all the individuals were relatively young and all were premenopausal. Groups were well matched for ethnicity, general adiposity (BMI, total fat mass, VAT, circulating level of leptin and adiponectin), fat distribution (waist to hip ratio [WHR], android and gynoid fat mass, percentage of leg fat), ectopic fat mass accumulation (liver fat and intramuscular fat), and dyslipidemia (triglycerides, cholesterol/HDL).

Consistent with the literature, the PMOS group showed signs of IR, demonstrated by a trend of higher fasting glucose and HOMA-IR (Table 1), as well as significantly higher testosterone (total and free) and anti-müllerian hormone (AMH) levels as expected. Nine of the 15 patients with PMOS had reported polycystic ovaries at screening as detected by pelvic ultrasound, and irregular menstrual cycles. To directly compare insulin sensitivity between the 2 groups of women, we performed an OGTT (Figure 1B). The glucose level was higher in PMOS compared with CTRL at the beginning and at the end of the OGTT (Figure 1B). Insulin secretion during OGTT challenge was higher in PMOS compared with CTRL (AUC, *P* = 0.04) and circulating FFA was higher in PMOS (AUC, *P* = 0.05); the latter suggests a difference in the antilipolytic effect of insulin between both groups which is consistent with earlier work (23). The glucose level was significantly different between 2 groups (AUC, *P* < 0.01), but not after adjustment with baseline glucose level.

In PMOS, leptin and other cytokines have been hypothesized as local regulators of reproductive function, acting directly on the ovaries (24) and within the brain to stimulate gonadotropin secretion (25). However, when we measured a panel of circulating inflammatory cytokines and chemokines (IL-1b, IL-6, IL-8, IL-10, TNF-α, MCP-1) in the serum of CTRL and PMOS, only IL-8 was increased (Figure 1C) in PMOS, arguing against an alteration in circulating major cytokines playing a role in our PMOS population. The difference of circulating IL-8 was confirmed by ANCOVA after adjustment for age (*P* = 0.037) or BMI (*P* = 0.038) and remained of comparable magnitude after adjustment for WHR (*P* = 0.058) or all 3 covariates jointly (*P* = 0.051), suggesting an implication of IL-8 in PMOS independent of central obesity. For example, IL-8 can alter follicular development within the ovaries and impact hormone production.

Hypertrophic adipocytes are markers of unhealthy adipose tissue expansion (26, 27), and they have been associated with IR in obesity (28). We determined the average adipocyte diameter on histological section of ABD and GF-AT in both groups (Table 1). GF adipocytes were on average larger than the ABD adipocytes in PMOS, but this was not observed in CTRL (Figure 1D).

A correlation matrix was generated to provide insight into the interrelationships of the lipids/obesity variables with adipocyte size in ABD- and GF-SAT (Figure 1E). In both depots, adipocyte size was positively correlated with adiposity markers (BMI, total fat mass, VAT mass, liver, and intramuscular fat) and diabetes risk markers (glucose, insulin, HgbA1c). Adipocyte size was also negatively correlated with the proportion of lower body fat and circulating levels of the adipokine adiponectin; the latter are markers of metabolically healthy adiposity (29). These results suggest that, in our study, adiposity is associated with larger adipocytes and a greater risk to develop metabolic syndrome (increase of ectopic fat accumulation and diabetes markers). The inverse correlation observed between adipocyte size and fat leg mass (FLM) suggests that women with a gynoid body type (with high FLM) have on average smaller adipocytes than the women with an android body type (characterized by low FLM). Importantly, our study includes a majority of women with an android body type (defined by WHR > 0.8), 19 android versus and 13 women with a gynoid body type (defined by WHR < 0.7), reflecting the fact that PMOS have greater central adiposity than the general population as previously reported (30, 31). Overall, these data suggest the influence of adipose tissue distribution in metabolic syndrome, including in the PMOS group.

### Upregulation of genes involved in adipogenesis and fatty acid regulation in all SAT cell types in PMOS compared with CTRL.

To identify key cellular composition and transcriptional differences between PMOS and CTRL groups in both ABD and GF-AT, we performed adipose single-nuclei RNA-seq (snRNA-seq). We used ICELL8 Smartseq full length technology, as previously described (32, 33). This study was performed on a subset of the larger population; 5 healthy women (CTRL) and 5 women with PMOS.

**Figure 2:**
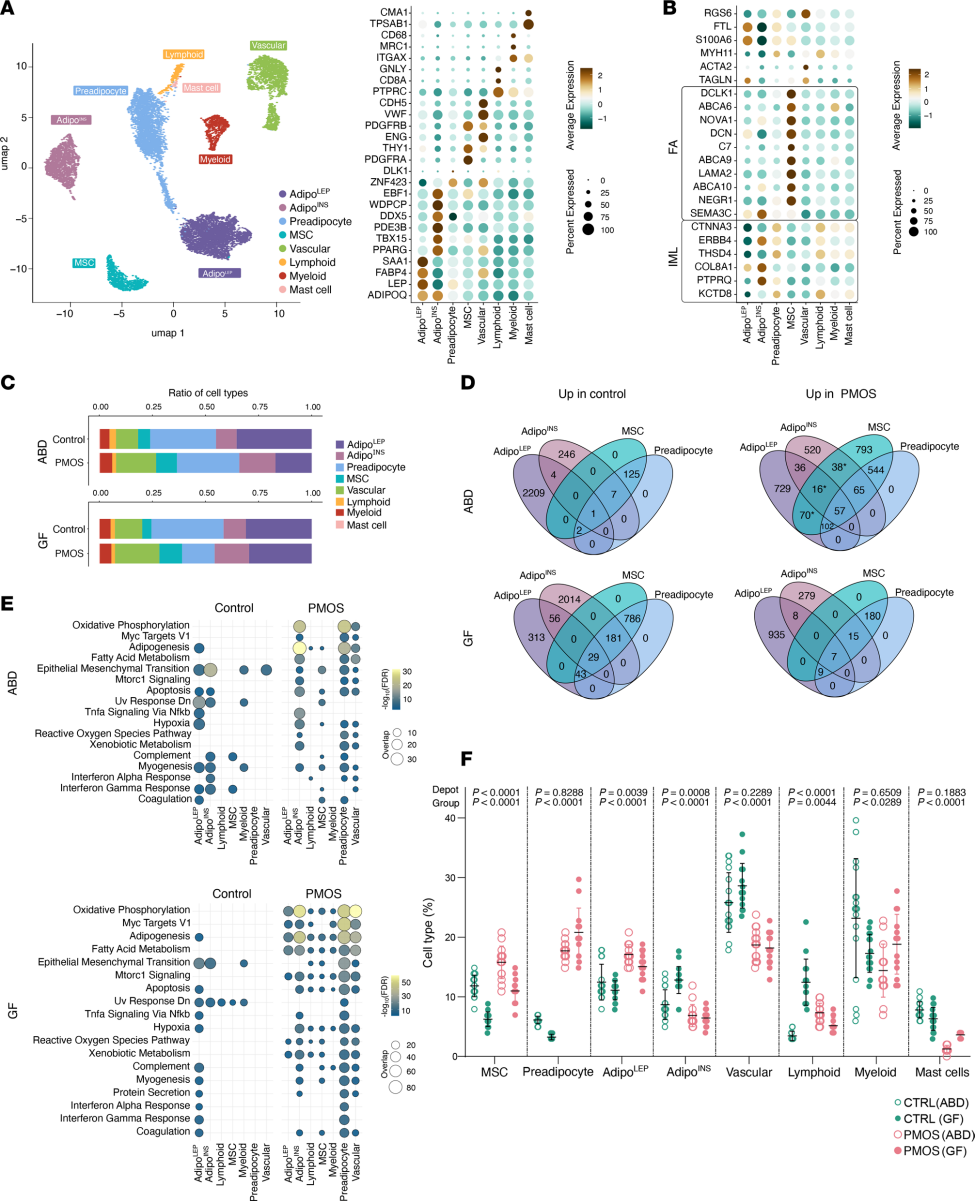
A single nuclei atlas of ABD and GF-AT in women with PMOS and BMI-matched women without PMOS. (**A**) UMAP projection of clusters formed by 11,638 human single nuclei isolated from ABD and GF-AT. *n* = 5 PMOS and 5 CTRL. The dotplot shows the marker genes used to identify each cluster. (**B**) Dotplot representation of relative expression of genes identified by Strieder-Barboza et al. (34) in each cell cluster. FA, Fibro Adipogenic Adipose Stem Cell; IML, Inflammatory Mesothelial like Adipose Stem Cell (34). (**C**) Stacked bar charts show the repartition of each cluster in CTRL and PMOS samples, split by depot (ABD and GF). (**D**) Intersection plots show the genes upregulated in CTRL and in PMOS that are common or unique to each cluster (MSC, preadipocytes, adipocytes). The analysis was performed independently in ABD (top graph) and GF-AT (lower graph). (**E**) Dotplot representation of functional enrichment analysis using Hallmark on the genes differentially regulated between CTRL and PMOS in each cell clusters. Pink indicates the pathways enriched in PMOS samples; green indicates the pathways enriched in CTRL samples. See also Supplemental Figure 1. Overlap represents the number of genes in pathways. (**F**) Scatter plot showing the proportions of each cell clusters in ABD and GF-AT estimated by deconvolution analysis using RNA-seq data from 17 CTRL women and 15 women with PMOS (including the 10 subjects used for snRNA-seq analysis). See also Supplemental Figure 1.

In total, 13,633 sequenced nuclei were included in our analysis. After quality filtering, we generated a library of harmony-integrated snRNA-Seq dataset that contained 11,638 single nuclei (5,557 from ABD-SAT and 6,081 from GF-SAT). Unsupervised clustering of the 11,638 nuclei revealed 8 uniquely defined clusters (Figure 2A), as reported previously in SAT using the same technology (icell8) (33). The dotplot Figure 2A shows the relative expression of the marker genes used to identify each cell cluster. The separation of preadipocyte and MSC clusters was made using well-known mesenchymal markers (Figure 2A) and markers highlighted by Strieder-Barboza et al. such as *DCLK1*; *ABCA6*, -*9*, and -*10*; *DCN*; *LAMA2*; *ERBB4*; *THSD4*; and *PTPRQ* (Figure 2B) (34). They also separated the adipose precursors (or ASCs) into 2 distinct subpopulations: the fibro adipogenic ASC (FA-ASC) and the inflammatory mesothelial like ASC (IML-ASC). Interestingly, they also showed adipocytes derived from these separate subpopulations manifest distinct metabolic phenotypes in vitro. In our dataset, based on the gene expression profiles, preadipocytes matched with their IML-ASCs, whereas MSC as defined here matched with their FA-ASCs (Figure 2B).

Interestingly, we identified 2 adipocyte populations that are transcriptionally similar to our previous snRNA-seq dataset of ABD s.c. WAT (33). These 2 adipocyte populations also have similar patterns of expression as 2 populations described in a separate study by Bäckdahl et al. using spatial transcriptomics (35). The authors showed that the adipocyte population expressing higher level of leptin (LEP) and serum amyloid A (SAA) was less insulin sensitive than another adipocyte population that expressed higher perilipin 1 (PLIN1) and also displayed a more robust transcriptional response to insulin. To stay consistent with this published literature, we decided to name the matching populations we identified in our dataset AdipoINS (for adipocyte expressing higher level of genes involved in insulin pathway) and AdipoLEP (for adipocytes expressing higher level of leptin). (Figure 2A). A differential gene expression comparison of AdipoINS versus AdipoLEP revealed, as expected, that the AdipoLEP are enriched for extra cellular matrix (ECM) genes such as *DCN*, *LUM*, *COL1A1*, *COL1A2*, and *MGP* as well as inflammatory genes such as *C1QC*, *CXCL14*, and *APOD* (Supplemental Figure 1, B and C; supplemental material available online with this article; https://doi.org/10.1172/jci.insight.199981DS1). To determine if one of these adipocyte populations might be hypertrophic, we evaluated the expression of 46 genes upregulated in adipocytes associated with hyperplasia in AdipoINS and AdipoLEP as identified by Gao et al. to be related to adipocyte morphology (hypertrophy versus hyperplasia) (36). We found that 32 genes (70%) that were elevated in hyperplastic adipose tissue were also upregulated in AdipoINS compared with AdipoLEP. This suggests that AdipoINS adipocytes are connected with hyperplasia rather than hypertrophic adipose tissue expansion (Supplemental Figure 1D).

The overall proportions of each population of cells were not different between adipose tissue depots (Figure 2C) or adipose distribution (Supplemental Figure 1E). Overall, there were no differences in cell-type proportions between CTRL and PMOS groups (Figure 2C).

The 8 gene clusters from the snRNA-seq analysis were then analyzed for genes up- or downregulation in PMOS using differentially genes expression (DEG) analysis between groups (CTRL versus PMOS) within each cluster. We intersected the list of DEG obtained per cluster and found that all the genes differentially expressed in preadipocytes were similarly affected in MSC, suggesting that these 2 populations are related; however, the genes dysregulated in the 2 adipocyte populations were unique to adipocytes, highlighting the difference between them (Figure 2D). Interestingly, we observed an exception to this pattern in the ABD depot where 10% of the genes upregulated in adipocytes from PMOS (AdipoINS and AdipoLEP) were also upregulated in MSC-PMOS (Figure 2D). Pathway analysis with the list of DEG in each cluster revealed that genes related to adipogenesis, fatty acid metabolism, and oxidative phosphorylation were uniquely upregulated in PMOS preadipocytes, adipoINS, and vascular cells (Figure 2E). These results were consistent in both depots. Additional pathways related to cell proliferation (Myc Target V1, Mtorc1) were upregulated only in clusters from PMOS GF depot (Figure 2E).

Next, to assess if the proportion of the 8 cell clusters identified by snRNA-seq (Figure 2A) differed between PMOS and CTRL in the larger group of 17 CTRL and 15 PMOS, we utilized our dataset as a reference to estimate cell-type proportions in bulk-RNA sequencing data obtained from the ABD and GF-SAT using our optimized deconvolution pipeline (37). This analysis revealed that the relative abundance of MSC, preadipocyte, and AdipoLEP are higher in PMOS compared with CTRL in both ABD and GF-SAT depots (Figure 2F). In contrast, we observed a decrease in the relative abundance of AdipoINS and vascular cells in the PMOS samples (Figure 2F). We also observed a decrease of the relative proportion of mast cells in both SAT depots in PMOS and an increase in the myeloid and lymphoid populations in both depots (Figure 2F).

### Testosterone level is negatively associated with healthy adipose tissue expansion.

**Figure 3:**
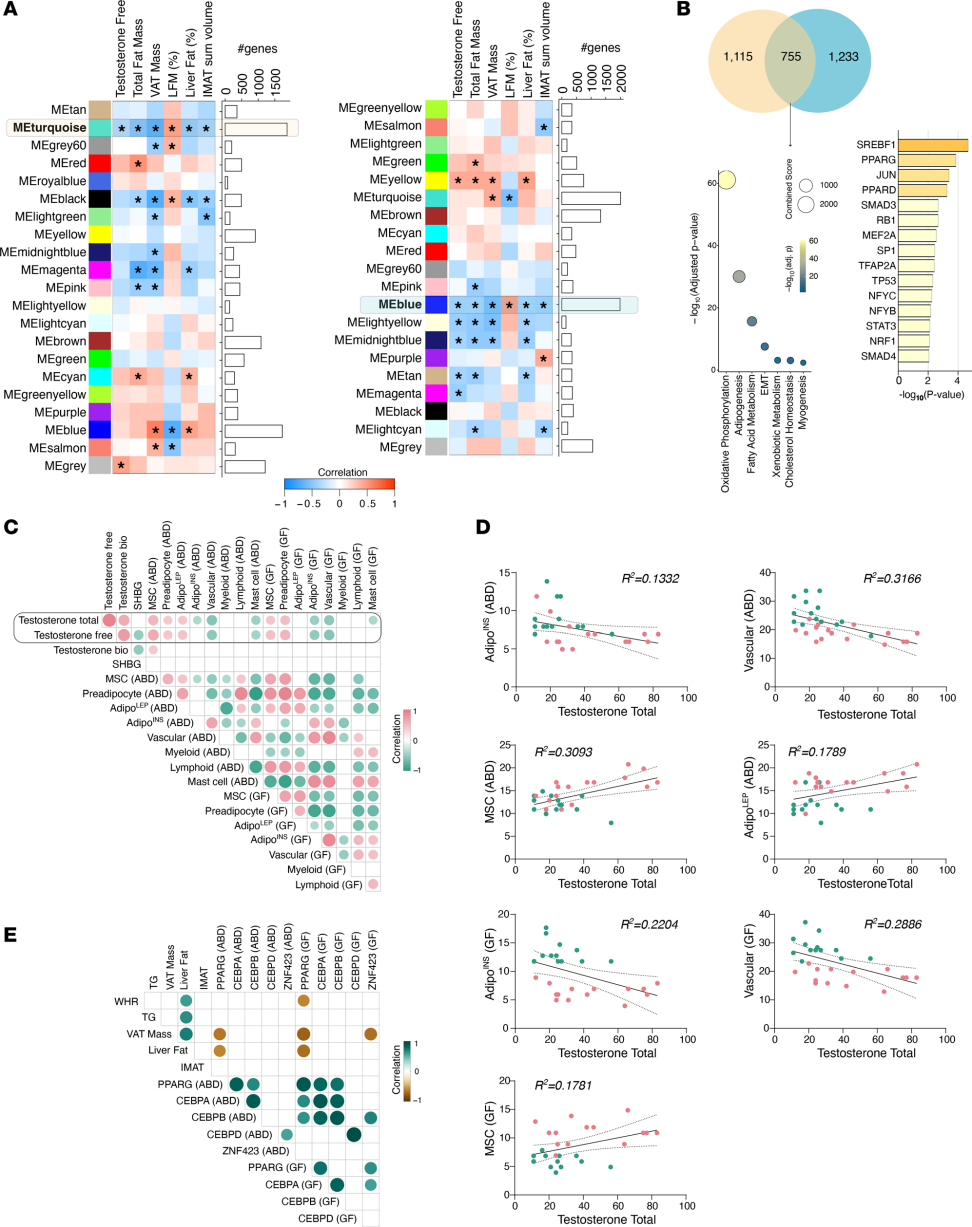
Association between testosterone level and SAT gene expression and cell population proportion in ABD and GF-AT. (**A**) Heatmaps representation of the correlations between the gene modules identified by WGCNA analysis and relevant clinical parameters in ABD and GF-AT. (**B**) Intersection plot showing the number of common genes between the turquoise module (in ABD-AT) and the blue module (in GF-AT). Pathway analysis reveals transcription factor (TRRUST) and pathways enriched in these common genes. EMT, Epithelial Mesenchymal Transition. (**C**) Correlation matrix of cell proportions and testosterone (total, free and bioavailable) and SHBG level. Only significant correlations (*P* < 0.05) are represented by a dot; negative correlations are depicted in green and positive correlations are depicted in pink. The size of the dot indicates the R coefficient. (**D**) Spearman correlation plots between total testosterone and relevant cell clusters in ABD and GF-AT depot, in combined group. PMOS participants are represented in pink; CTRL participants are represented in green. See also Supplemental Figure 2. (**E**) Correlation matrix of ectopic fat accumulation markers and adipogenesis-related genes in ABD and GF-SAT depot. Only significant correlations (*P* < 0.05) are represented by a dot; negative correlations are depicted in brown, and positive correlations are depicted in green. The size of the dot indicates the R coefficient.

To investigate the influence of sex hormones on the metabolic phenotype of the individuals, we analyzed the relationship between the level of testosterone (free and bioavailable), sex hormone binding globulin (SHBG), anti-mullerian hormone (AMH), and luteinizing hormone and Follicle-stimulating hormone ratio (LH:FSH) as well as the clinical data. Only the levels of androgen hormones were correlated with these clinical traits (Supplemental Figure 2A). To further investigate the role of testosterone on SAT gene expression and cell composition, we performed RNA-seq on snap-frozen biopsies of ABD and GF adipose tissue. We first weighted gene coexpression network analysis (WGCNA) using adiposity markers and systemic testosterone level as variables. This analysis revealed 21 and 20 coexpression networks in ABD and GF-SAT, respectively (Figure 3A). One gene expression network (or module labeled ME on Figure 3A) in each depot (MEturquoise in ABD and MEblue in GF) was significantly positively correlated with testosterone, total fat mass, VAT mass, liver, and intramuscular fat accumulation and negatively correlated with the percentage of leg fat mass (LFM) (Figure 3A). These 2 networks have 755 common genes, representing around 40% of the total genes included in the individual network. Pathway and transcription factor analysis of these common genes identified “Adipogenesis” and “Oxidative phosphorylation” as the top differentially regulated pathways (Figure 3B). The transcription factors identified as targeting these genes are previously known as critical transcription factors controlling adipogenesis (PPARG) and fatty acid/cholesterol biosynthesis (SREBF1). These results demonstrate that testosterone is associated with the transcriptional regulation of adipogenic genes in both ABD and GF depots and suggest that it is responsible for excess fat accumulation. The pathway analysis generated from the unique genes in each network are reported in Supplemental Figure 2B. We highlight “adipogenesis” is present only in the GF network, suggesting that genes involved in adipogenesis and that participate in whole-body fat accumulation only have an effect in the GF depot. The correlations of each gene expression with key clinical parameters are shown in Supplemental Table 1. This negative correlation suggests that testosterone is associated with a reduction of healthy adipose tissue expansion (negative correlation with tissue vascularization and insulin sensitive adipocytes).

Next, we analyzed at the associations between the clinical parameters, specifically testosterone level, and the relative proportion of each cell cluster in the snRNA-seq data set (Figure 2B). The correlation matrix revealed a positive correlation between the level of T (total and free) and the relative proportion of precursor cells (MSC and preadipocytes) in both depots and only in ABD depot a positive correlation between T and the relative proportion of AdipoLEP. Interestingly, an inverse correlation was observed between T level (total) and the relative proportion of AdipoINS and vascular cells in both depots (Figure 3C). Figure 3D shows the individual correlations used for generating the correlation matrix in Figure 3C between T-Total and cell proportions. The correlation matrices generated in only ABD and only GF-SAT are reported in Supplemental Figure 2C. Interestingly, the expression of androgen footprint gene set (*AKR1C1*, *AKR1C2*, *AKR1C3*, *CYP19A1*, *HSD17B1*) is higher in adipoLEP compared with adipoINS (Supplemental Figure 2D). AdipoLEP proportion is higher in PMOS versus CTRL. These observations suggest that adipoLEP in PMOS are actively processing androgen, potentially participating in the local effect of androgen on adipose tissue.

In both depots, we found that the relative proportion of AdipoINS to vascular cells was negatively associated with total adiposity markers (BMI, total fat mass) and central obesity markers (WHR, VAT mass, liver fat) (Supplemental Figure 2C). These data suggest that, locally, AdipoINS and the vascular compartments play a protective role to limit central adiposity and ectopic fat accumulation, potentially by favoring vascularization along with healthy SAT expansion in both SAT depots. This is consistent with the expandability hypothesis (38).

To validate the hypothesis that testosterone limits the expansion of SAT in PMOS, leading to the ectopic accumulation of fat, we explored the relationship between the expression of adipogenesis related genes in the ABD and GF-SAT against TG level, VAT mass, liver fat, IML, and WHR (39). In alignment with our earlier findings, we found negative correlations between the expression of the master regulator of adipogenesis, *PPARG*, in ABD and GF-SAT with VAT mass and liver fat accumulation (Figure 3E).

### DEG pseudotime analysis suggests a block to differentiation in PMOS preadipocytes.

Next, we leveraged the use of previously validated pseudotime trajectory analyses and RNA velocity in silico. Pseudotime analysis reveals the interactions of cells through commitment, differentiation, and maturation along developmental pathways. We used this method to track adipocyte differentiation from the 2 precursor populations (MSC and preadipocytes) into the 2 adipocyte populations comparing the results between the CTRL and PMOS groups.

**Figure 4:**
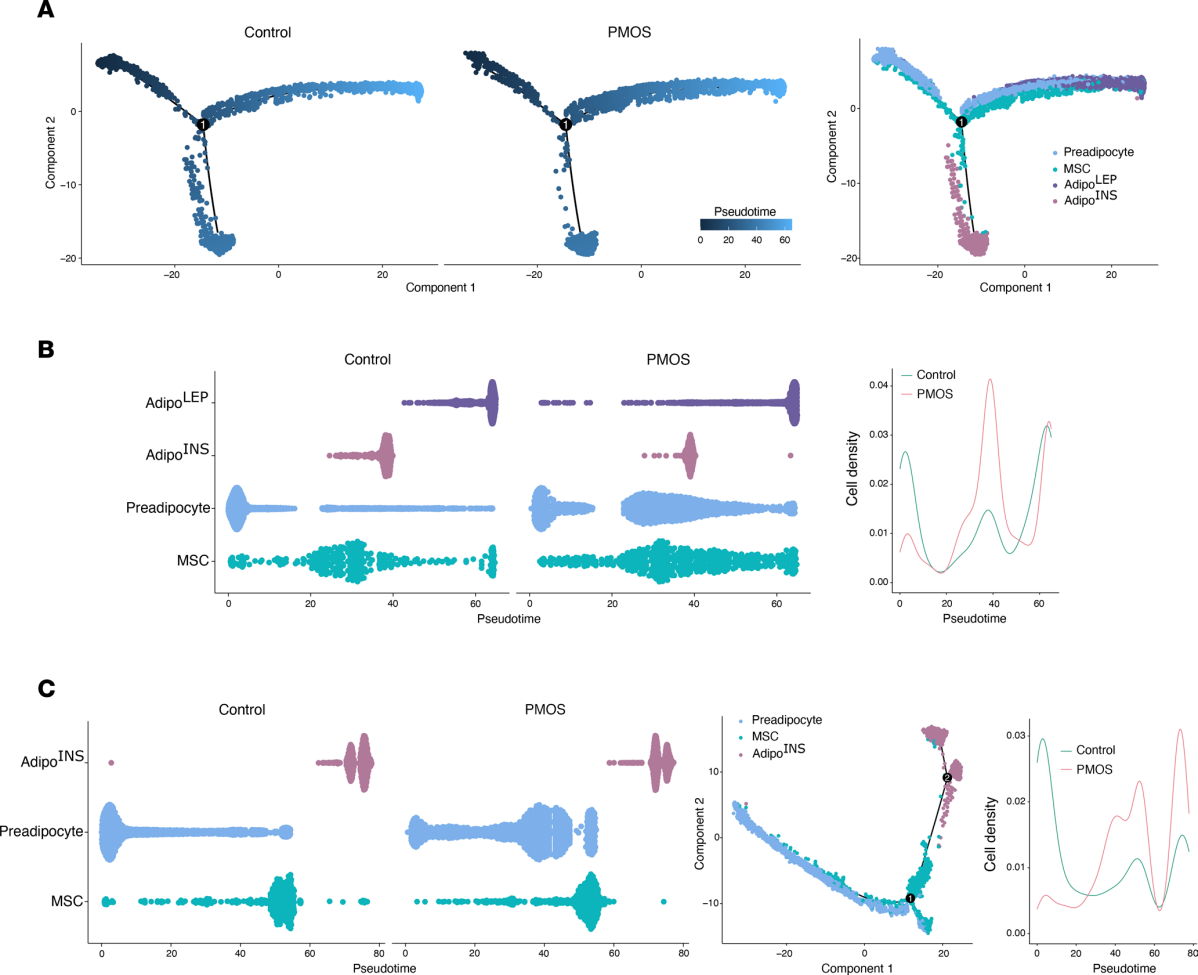
Pseudotime analysis suggests block of differentiation in PMOS. (**A**) Distinct state of cells identified by pseudotime analysis in CTRL and in patients with PMOS using MSC, preadipocytes, AdipoLEP, and AdipoINS. (**B**) Ordering and quantification of cells from different clusters identified in **A** along the pseudotime trajectory and according to the condition (CTRL or PMOS). (**C**) Distinct state of cells identified by pseudotime analysis in CTRL and in patients with PMOS using MSC, preadipocytes and AdipoINS only.

Surprisingly, the pseudotime trajectory initiated in the preadipocyte cluster and not at the MSC cluster. The model predicted a split into 2 branches: one continued into the AdipoLEP cluster, while the other one continued toward the AdipoINS cluster (Figure 4A) in both clinical groups (Figure 4A). The quantification of cells according to their position in the projected pseudotime pathway indicates that preadipocytes in the PMOS group accumulate in the middle of the pseudotime pattern as compared with CTRL (Figure 4B). This result is consistent with a block in adipocyte differentiation in the PMOS preadipocytes.

When we reanalyzed the dataset using only the MSC, preadipocytes, and AdipoINS, we obtained similar results (Figure 4C), with a trajectory initiated in preadipocytes rather than in MSC and accumulation of preadipocytes in the middle of the pseudotime pathway only in the PMOS group. These distinct patterns and differences between CTRL and PMOS suggest that the MSC and preadipocytes likely represent 2 distinct precursor cell types and are not part of a continuum. Also, comparison of PMOS to CTRLs suggest that preadipocytes but not MSC fail to differentiate fully into AdipoINS adipocytes in PMOS.

### Accumulation of MSC in the SVF of patients with PMOS.

**Figure 5:**
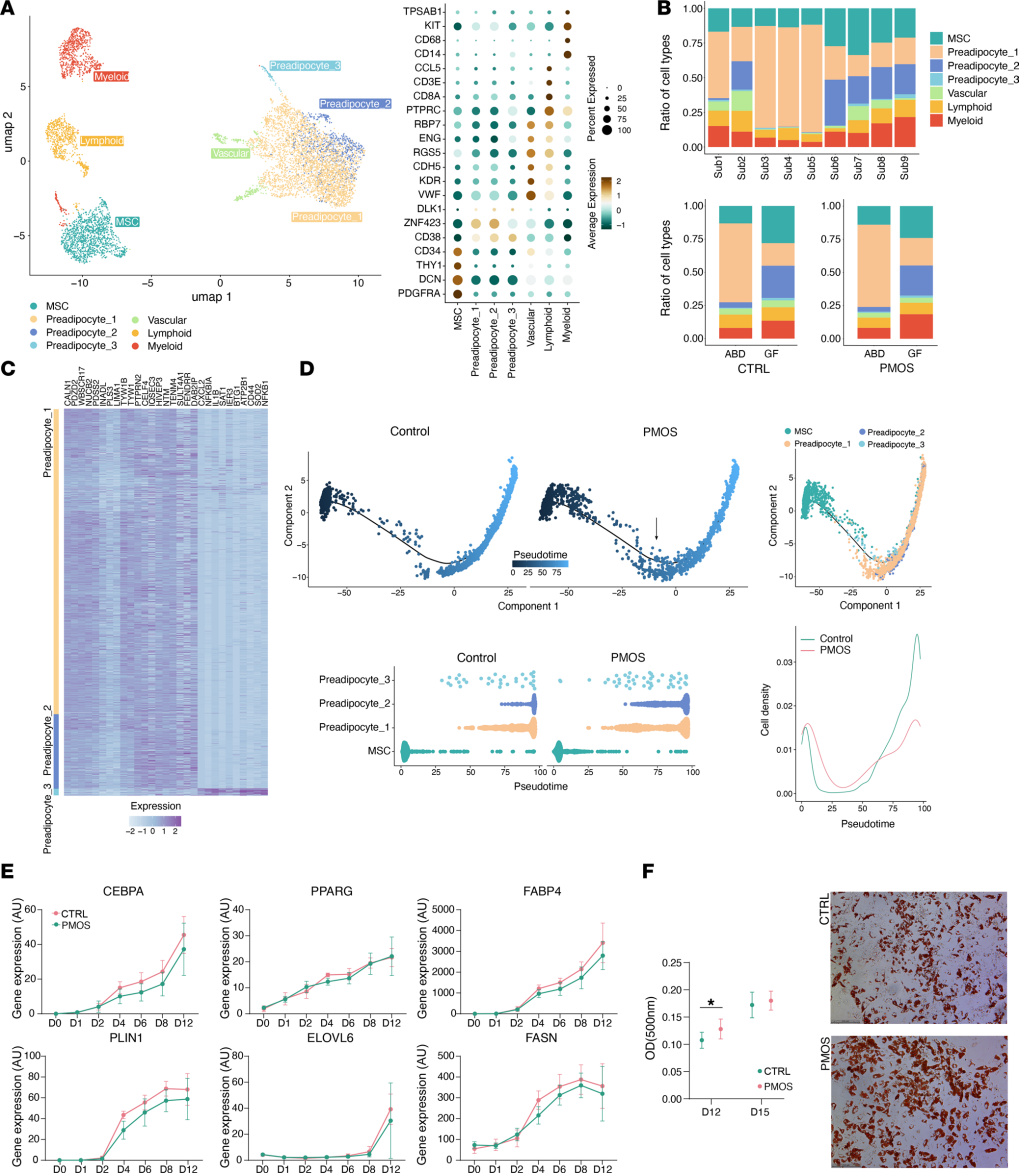
A single-cell ATLAS of ABD and GF-stroma vascular fraction in women with PMOS and BMI-matched CTRL. (**A**) UMAP projection of clusters formed by 7,204 human single cells extracted from ABD and GF-SVF. *n* = 5 CTRL and 4 PMOS. The dotplot shows the marker genes used to identify each cluster. (**B**) Stacked bar plots showing the proportion of each cluster per subject and their average per group (CTRL versus PMOS) and per depot (ABD versus GF). (**C**) Heatmap showing the expression of the most DEG between the 3 populations of preadipocytes in each cell (Only Pread-1, Pread-2, and Pread-3 being included on the heatmap). (**D**) Distinct state of cells identified by pseudotime analysis in CTRL and in patients with PMOS. Ordering and quantification of cells from different clusters identified in **A** along the pseudotime trajectory and according to the condition (CTRL or PMOS). (**E**) Gene expression level during 12-days of adipogenic differentiation of ABD-MSC isolated from CTRL-SVF (*n* = 5) and PMOS-SVF (*n* = 5). (**F**) The degree of differentiation was measured by Oil red O in each group after 12 days (D12) and 16 days (D16) of differentiation. A representative picture of cells isolated from CTRL-SVF and from PMOS-SVF after 12 days of differentiation is shown. See also Supplemental Figures 3 and 4. Original magnification, ×20. *P < 0.05 Mann Whitney test between group.

The frozen tissue snRNA-seq analysis suggested a potentially novel hypothesis for metabolic dysregulation in PMOS — specifically, a block in precursor differentiation into mature adipocytes. To further test this hypothesis, we performed scRNA-seq on freshly isolated SVF from ABD and GF adipose tissue in *n* = 4 individuals with PMOS and *n* = 5 CTRL from our original larger cohort. A total of 7,204 sequenced cells were included in our analysis. We identified 7 clusters and as above we named them according to the relative expression of the known markers of MSC, preadipocytes, vascular, lymphoid, and myeloid cells (Figure 5A). The marker genes used are reported in the dotplot in Figure 5A. Additional marker genes of MSC are shown in Supplemental Figure 3B, demonstrating a clear distinction between MSC and preadipocytes, where expression of stem cell marker genes is absent in the preadipocytes. We found a similar distinction between the MSC and preadipocyte fractions, which we have previously identified in our snRNA-seq dataset, notably the MSC cluster appears to be more fibro-adipogenic (Supplemental Figure 3C). We also observed that the preadipocytes were mainly in the S and G2M phases of the cell cycle, in both depots, indicative of their highly proliferative state (Supplemental Figure 3D).

We next quantified the relative cell proportion in the SVF of each depot and in each group. In PMOS, the relative proportion of MSC was higher, whereas the relative proportion of preadipocyte populations was lower (Figure 5B). Importantly a unique preadipocyte population was identified in PMOS, preadipocyte 3; however, very few cells were identified in this cluster (Figure 5B). DEG analysis between the 3 preadipocyte populations revealed that the third preadipocyte cluster enriched in PMOS displayed a unique transcriptome with higher expression of inflammatory genes compared with the 2 other preadipocyte populations (Figure 5C and Supplemental Figure 3E). Further pathway analysis of the unique genes in each preadipocyte cluster showed that 1 cluster of preadipocytes upregulated in PMOS group (Preadipocyte 2) expressed higher levels of genes encoding extracellular matrix proteins including ECM glycoproteins, collagens, and proteoglycans (Supplemental Figure 3F). Preadipocyte 1 cluster (up in CTRL group) showed an increase of genes involved in adipogenesis (*FTL*, *CXCL14*, *FABP4*, *CFD*) and in G2M checkpoint genes, for example TERT (Supplemental Figure 3G). This signature suggests chromosomal stability by maintaining telomere length allowing cells to avert senescence. Taken together, these gene expression patterns is consistent with preadipocytes in the CTRL group that are highly proliferative and have a higher adipogenic capacity than the preadipocytes in PMOS; the latter being more fibrotic and proinflammatory.

To test if the 3 preadipocyte populations and MSC are part of a continuum, we included them in a second pseudotime trajectory analysis. This revealed the expected continuous gene regulatory program starting with MSCs and finishing at the preadipocyte lineage (Figure 5D). This is consistent with our earlier observations in full SAT tissue where there is an accumulation of preadipocyte 1 cells in the PMOS group. These cells are in the middle of the pseudotime pathway (indicated by an arrow of Figure 4D). We interpret this finding as consistent with the lack of capacity to differentiate along the continuum. More importantly, a larger peak of cells at the end of the pseudotime in CTRL versus PMOS is consistent with more cells engaged in terminal adipogenesis in CTRL as compared with PMOS.

To further evaluate the potential block in differentiation in PMOS more directly, we compared the level of differentiation of the progenitors from PMOS versus CTRL in vitro using ADSC cultured over several days. As shown by Oil red O quantification and the level of expression of expression of adipogenic marker genes at the beginning of the differentiation (*CEBPA* and *PLIN1*), the PMOS cells differentiated more than the CTRL (Figure 5E for ABD depot and Supplemental Figure 4 for GF depot). The difference was not maintained when the cells were allowed to continue to differentiate for 16 days instead of 12 days (Figure 5F). To summarize, PMOS SVF contains more MSC than the CTRL-SVF and these MSC are more adipogenic.

### SLIT2 secretion is increased in MSC in PMOS and could block preadipocyte differentiation via its interaction with ROBO1/2.

**Figure 6:**
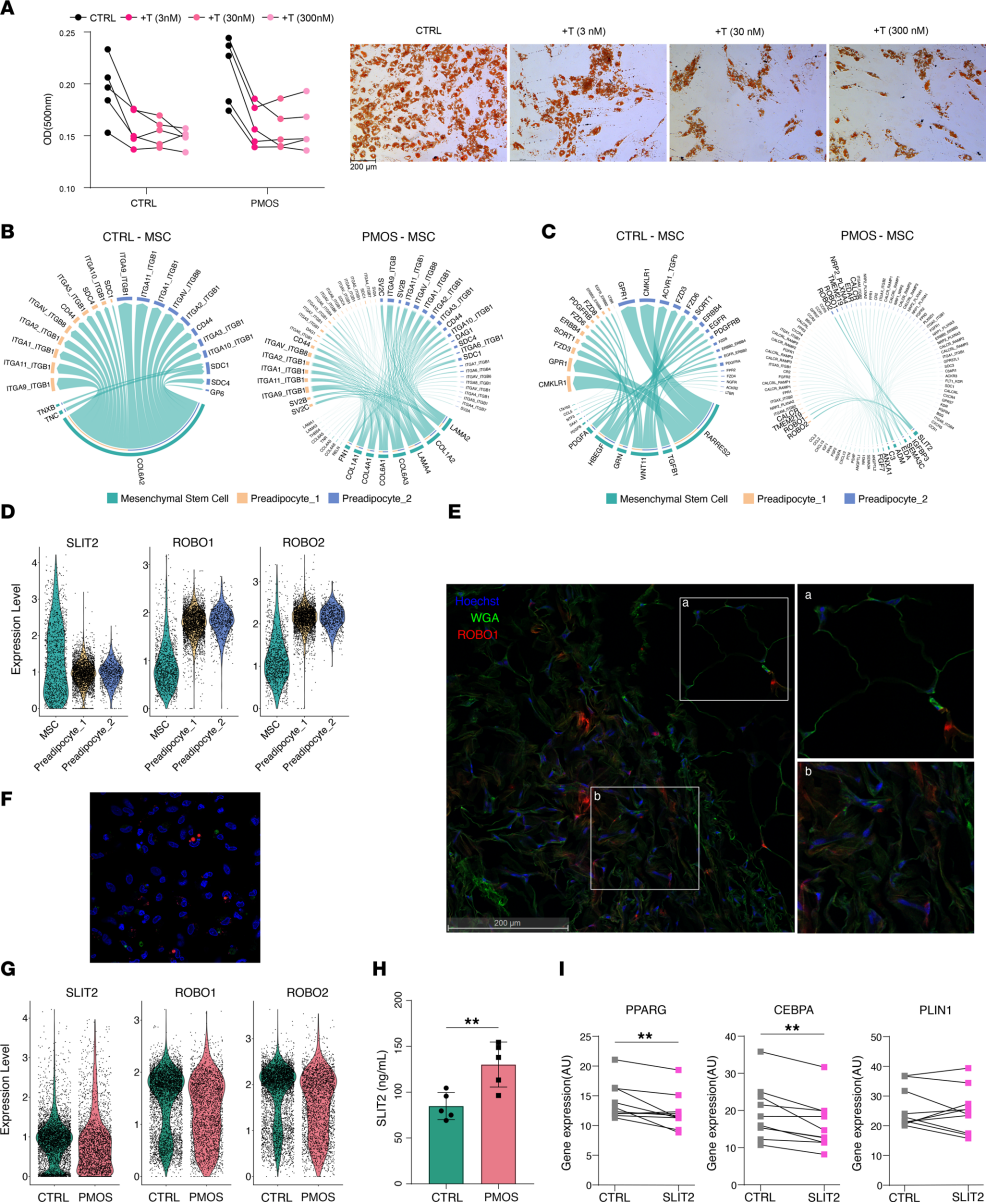
Potential interaction between MSC and Preadipocytes via SLIT2/ROBO pathway. (**A**) Level of differentiation of ABD-ADSCs after 12 days of incubation of adipogenic differentiation cocktail supplemented with testosterone (T). Oil red O was used to quantify the lipid accumulation at day 12. *n* = 10 ADSCs, isolated from 5 CTRL and 5 PMOS. Representative pictures of CTRL ADSCs at D12 of differentiation with or without testosterone in the media. Scale bars: 200 μm. (**B**) Circle plots of secreted signaling pathways coming from MSC population enriched in CTRL and in PMOS group. The colors of the circle specify which cell type receives the signal. (**C**) Circle plots of ECM-receptor signaling pathways enriched in MSC CTRL and in MSC PMOS group. The colors of the circle specify which cell type receives the signal. (**D**) Violin plots comparing the expression level of *SLIT2*, *ROBO1*, and *ROBO2* in MSC, Pread-1, and Pread-2 nuclei between CTRL and PMOS group. (**E**) Immunofluorescence staining of s.c. adipose tissue using WGA (488 excitation length of the fluorochrome) and ROBO1 (647 excitation length of the fluorochrome) antibodies. Hoescht was used to stain the nuclei. Scale bar: 200 μm. (**F**) Immunofluorescence staining of stroma vascular fraction cells using ZNF423 (488 excitation length of the fluorochrome) and ROBO1 (647 excitation length of the fluorochrome) antibodies. Hoescht was used to stain the nuclei. Original magnification, ×20. (**G**) Violin plots comparing the expression level of *SLIT2*, *ROBO1*, and *ROBO2* in scRNA-seq dataset. (**H**) Histogram of SLIT2 secretion in 24-hour-condition media of MSC isolated from CTRL (*n* = 5) and from PMOS (*n* = 5). **P < 0.05 by Mann-Whitney *U* test. (**I**) Expression of *PPARG*, *CEBPA*, and *PLIN1* in cells treated with SLIT2 one day before the beginning of differentiation and during 4 days of differentiation. Cells were collected at day 4, RNA was extracted, and gene expressions determined by qPCR. See also Supplemental Figure 5. **P < 0.01 by Wilcoxon matched-pairs signed rank test.

We hypothesized that, even though their proportion is higher, the MSC population observed in PMOS might not differentiate properly due to an inhibitory microenvironment in vivo. One possible inhibitor could be testosterone itself because it is elevated in PMOS. Thus, we first tested this hypothesis during human adipogenesis by adding testosterone continuously into the differentiation media of cultured MSC. Consistent with a prior published report (40, 41), the addition of T resulted in a decrease in MSC differentiation from both depots of CTRL and PMOS (Figure 6A). These results suggest that endocrine signal(s) like testosterone might reduce the capacity for differentiation of APCs in PMOS in vivo. To search for additional potential paracrine pathways that might operate between MSC and preadipocytes in PMOS and CTRL, we went back to the results of our CellChat analysis of our scRNA-seq dataset. This analysis highlighted a large number of putative secreted signals (Figure 6B) and ECM-receptor signals (Figure 6C) originating from MSC that connect to preadipocytes 1 and 2 that were predicted to occur in PMOS but were not observed in CTRL.

The number of secreted signals originating in preadipocyte 1 was also higher in the PMOS group (Supplemental Figure 5A). Of these signaling pairs, SLIT2/ROBO1/2 was predicted as the strongest pathway between MSC and preadipocytes in PMOS (Figure 6C). Interestingly, the expression of SLIT2 was higher in MSC and expression of ROBO1 and ROBO2, the cognate receptors for SLIT1/2, were higher in preadipocytes (Figure 6D and Supplemental Figure 5B). We confirmed that preadipocytes expressed ROBO1 protein at their surface by costaining SVF cells with ZNF423 (marker of committed preadipocytes) and ROBO1 (Figure 6, E and F, and Supplemental Figure 5C). We also confirmed that ROBO1 protein was expressed in adipocytes (Supplemental Figure 5D). The level of expression of *SLIT2* and *ROBO1/2* mRNAs were similar between CTRL and PMOS, both in our scRNA-seq data set (Figure 6G) and during differentiation (Supplemental Figure 5E). Importantly, MSC isolated from PMOS ABD biopsies secreted greater level of SLIT2 protein compared with MSC isolated from CTRL ABD biopsies (Figure 6H, 24-hour secretion) and the expression of *SLIT2* decreased sharply after initiation of differentiation in both CTRL and PMOS cells (Supplemental Figure 5E). To determine the relevance of these results, we added SLIT2 early during in vitro differentiation (between day –2 and day 4) and found that SLIT2 recombinant protein reduced the expression of key adipogenic genes (*CEPBA*, *PPARG*, *PLIN1*; Figure 6I). Altogether, these data reveal that SLIT2 secreted by MSC might reduce the adipogenic capacity of neighboring preadipocytes within SAT. Because there is a higher proportion of MSC in PMOS and there is a higher level of secretion of SLIT2 by MSC from PMOS, this pathway may explain the observed “block” in adipocyte differentiation and differences in mature adipocyte populations in PMOS.

## Discussion

Alterations of SAT structure and transcriptome have been observed in metabolic diseases such as T2D. It has never been studied in PMOS, syndrome associated with hormonal unbalance and in majority of the cases associated with IR. In this translational study, we combined deep whole-body phenotyping with advanced cellular profiling of ABD and GF SAT to investigate their phenotype in PMOS pathogenesis.

The 2 groups of women studied could be categorized as obese with central fat accumulation and mild metabolic syndrome features; the most striking differences between the 2 populations (besides the PMOS diagnosis) being the high levels of testosterone and AMH in the PMOS group. In this context, our study could be used as a potentially new model to dissect adipose pathology in elevated testosterone environment.

RNA-seq revealed an alteration of adipogenesis and fatty acid metabolism gene expressions in both SAT in PMOS, suggesting impaired SAT expansion in PMOS. Using snRNA-seq, we defined for the first time the cellular composition of ABD and GF-SAT in PMOS and found an increased proportion of fibrotic adipocytes (AdipoLEP) and a reduction of insulin-sensitive adipocytes (AdipoINS), 2 subpopulations of adipocyte identified by previous studies. The decrease of the hyperplastic like AdipoINS (36) subpopulation in the PMOS group (more IR) corroborates previous findings that ABD-SAT is hypertrophic in T2D compared with non-T2D and that fat cell size correlated positively with IR (42).

Historically, SAT has been widely described as a metabolic sink serving as buffer to avoid multiorgan metabolic effects of weight gain (43, 44). However, growing number of studies using snRNA-seq and scRNA-seq revealed the importance of not only the VAT and ectopic fat deposition in other organs in metabolic risk, but also the heterogeneity within SAT, both at cell-type composition and at the molecular level (adipocyte function and size) (35, 45–48). Specifically, a recent bulk RNA-seq study showed strong concordance of the IR transcriptome between SAT and VAT mass, and most IR-related gene expression patterns reversed after surgical weight loss (49). These findings highlighted a dynamic and reversible transcriptional landscape of IR in SAT, shared in part with VAT. Our work aligned with these results, showing the importance of SAT cellular composition (notably ratio of AdipoINS/AdipoLEP) and transcriptome in SAT expansion and potentially IR in PMOS. We found interesting associations with the metabolic syndrome IR score, calculated as described in ref. 50 and SAT biology (Supplemental Figure 6). Notably, we found positive correlation between IR score and adipocyte size in both depots and negative correlation between IR score and the relative proportion of AdipoINS and vascular cells in both depots. These findings suggest that adipose tissue alterations are early markers of metabolic syndrome features associated with PMOS.

Besides the accepted role of the adipocytes in AT expansion, our work established a new role of adipocyte precursor cells in AT expansion and metabolic syndrome. Pseudotime analyses indicated impaired preadipocyte differentiation in PMOS. We confirmed this hypothesis by using scRNA-seq on the SVF of the ABD and GF-SAT. We identified an alteration of the preadipocyte transcriptome in the PMOS versus CTRL samples, with increased expression of profibrotic and inflammatory genes in PMOS preadipocytes (51). We also observed an increased proportion of mesenchymal stem cells (MSC) in PMOS, with strong adipogenic potential in vitro, resembling fibroadipogenic progenitors previously described (34). While this appears inconsistent with impaired differentiation, the expansion of MSC may represent a compensatory attempt to preserve healthy SAT expansion in an adverse microenvironment. Earlier work (52), using a simple FACS method, did not allow the identification of MSC and preadipocytes in the same sample as we describe here using our unbiased scRNA-seq method on SVF (53). Specifically, our work detected potential interactions between these 2 unique populations that could play a critical role in SAT expansion. Cell-cell communication analysis (CellChat) predicted altered signaling between MSC and preadipocytes in PMOS, highlighting the SLIT2/ROBO pathway as a potential regulator of adipogenesis and inhibitor of adipogenesis in women with PMOS. While SLIT2 is known for its role in adipose tissue browning, our data suggests it also directly modulates preadipocyte differentiation in the context of PMOS. Importantly, we showed that SLIT2 secretion is higher in MSCs from PMOS compared with CTRL, establishing its unique role in MSC/preadipocyte interaction in PMOS. SLIT2 could also participate to block SAT expansion via its inhibitory action on angiogenic remodeling (54). It is noteworthy that SLIT/ROBO signaling has been shown to be involved in testosterone regulation in Leydig cell steroidogenesis (55). Taken together, our studies suggest SLIT/ROBO signaling may be a potential therapeutic target in PMOS, favorizing healthy SAT expansion and limiting ectopic fat accumulation.

Finally, the strong association between testosterone level and metabolic syndrome risk suggests a complex interaction between androgen hormones and other clinical outcomes in PMOS. The participants with PMOS enrolled in our study also showed signs of a reproductive phenotype, characterized by irregular menstruation, polycystic ovaries and high levels of sex hormones (testosterone and AMH). Importantly, we did not find any associations between ectopic fat accumulation or SAT cellular composition and AMH or LH:FSH levels, other sex hormones that are elevated in PMOS (56). Based on these observations, we evaluated the possible role of solely testosterone on SAT biology. Using a previously validated deconvolution pipeline, we showed that elevated testosterone in PMOS is associated with altered SAT cellular composition. Testosterone levels were negatively correlated with insulin-sensitive adipocytes (AdipoINS) and vascular cells and positively correlated with adipocyte precursor cells (MSC and preadipocytes). These findings suggest that, in PMOS, testosterone may promote precursor accumulation while impairing their differentiation, contributing to unhealthy SAT expansion. Although MSCs ex vivo display strong adipogenic potential, the PMOS microenvironment may mask this function. Further studies are required to investigate the role of testosterone and the discovery of new candidates that participate in this complex interaction would reveal ways to relieve the block on preadipocyte differentiation, promote healthy SAT expansion, reduce hypertrophic dysfunction, and limit ectopic fat deposition, IR, and cardiometabolic risk in PMOS.

In conclusion, our findings point to a complex, tissue-specific dysregulation of adipose development in PMOS, where impaired SAT expansion, altered adipocyte lineage balance, and adaptive but insufficient compensatory responses collectively contribute to metabolic dysfunction and potentially PMOS phenotype. Further studies are needed to translate these cellular changes into systemic metabolic consequences and to explore whether targeting adipose progenitor dynamics could ameliorate PMOS-associated metabolic risk and features.

## Methods

### Sex as a biological variable

We included only women in this work because the clinical markers used to define the PMOS group are directly tied to the female reproductive system. The findings are relevant only to this sex group.

### Participants and tissue collection

Thirty-two healthy premenopausal, weight-stable females aged 20–40 years with a body mass index (BMI) between 23 and 40 kg/m^2^ were recruited in Orlando, Florida, USA, using advertisements approved by the AdventHealth Institutional Review Board. Patients were excluded if they reported a history of chronic disease (diabetes, heart or liver disease, high blood pressure, and gastrointestinal disorder), recent weight loss or gain (>3 kg over the past 8 weeks), had abnormal blood or urine and clinical lab values, or used oral contraceptives or hormone replacement therapy. We excluded participants with any metabolic disorder; specifically, fasting plasma glucose ≥ 126 mg/dL, or HbA1c > 6% or a diagnosis with T2D, liver disease (AST or ALT > 2.5 times the upper limit of normal), kidney disease (creatinine > 1.6 mg/dL or estimated GFR < 60 mL/min), dyslipidemia (including triglycerides > 500 mg/dL, LDL > 200 mg/dL), uncontrolled hypertension (BP > 160 mmHg systolic or > 100 mmHg diastolic), history of bariatric surgery. We also excluded participant who uses oral or injectable antihyperglycemic agents (metformin, sulfonylureas, DPP IV inhibitors, SGLT-2 inhibitors, thiazolidinediones, acarbose, GLP-1 analogs, and insulin unless willing to undertake a washout period of 15 days for metformin and GLP-1 analogs) or uses any medications known to influence fat and/or energy metabolism (e.g., growth hormone therapy, glucocorticoids [steroids], etc.)

PMOS was defined based on medical records showing a diagnosis of PMOS, including: hyperandrogenism (clinical or biochemical) and ovarian dysfunction: oligo-anovulation OR ultrasound evidence of polycystic ovarian morphology when conditions that mimic PMOS were excluded. The CTRL women did not have any features of PMOS.

ABD-AT biopsies were collected with a 3-hole 2.5 mm liposuction cannula from the mid-abdomen approximately 5–8 cm lateral to the umbilicus. GF AT was collected 10–20 cm below the greater trochanter on the most lateral side of the upper thigh. Samples were cleaned at the bedside; a fraction of the sample was snap frozen in liquid nitrogen and a fraction of the sample was fixed overnight in Z-fix solution for future histology procedures. The remainder was immediately used for stromal vascular fraction (SVF) isolation as described in ref. 57. A portion of the freshly isolated SVF cells were used for scRNA-seq analysis for 9 randomly selected participants. The same participants were selected to perform snRNA-seq analysis on the frozen SAT biopsies. Detailed phenotyping of the participants included the following: anthropometric measures (weight, height, waist, hip, and thigh circumferences), fasting glucose, lipid, inflammation and hormone profiles, and body composition measured by dual-energy x-ray absorptiometry (DXA) using a GE Lunar iDXA whole-body scanner. Volumetric measurement of fat and other organs (liver, kidneys, heart, brain, skeletal muscle, and intramuscular AT) was completed using a Philips Achieva 3T magnetic resonance imaging/multinuclear magnetic resonance spectroscopy. Resultant images were analyzed using Analyze software 11.0 (Biomedical Imaging Resource, Mayo Clinic). Blood samples were collected the day before the adipose biopsies. All procedures were performed under a research protocol approved by the AdventHealth Institutional Review Board.

### Blood analysis

Fasting blood samples were analyzed in clinical chemistry laboratory at either Adventhealth Orlando or onsite at the Translational Research Institute. Before and during the OGTT, blood samples were collected on ice, centrifuged at 1,500 × *g*, aliquoted, and frozen at –80°C until further analyses. Glucose was monitored during the procedure using a Nova StatStrip glucose meter (Nova Biomedical). FFAs were measured by the NEFA-HR (nonesterified fatty acid HR series) assay (Fujifilm Wako Diagnostics) according to manufacturer’s protocol. Insulin was measured in undiluted human serum using the Human Insulin Kit (Meso Scale Diagnostics, K151BZC) (23) according to the manufacturer’s protocol. The homeostasis model assessment of the IR index (HOMA-IR) was calculated as follows: ([fasting insulin in mU/L] × [fasting glucose in mmol/L]/22.5). Data from the OGTT were used to calculate AUC to estimate the insulin sensitivity index. We used a repeated measures model (90, 120, 150, 180 minutes) with square root baseline as a covariate Chemokines and cytokines were measured in serum samples using U-plex adipokine Combo 1 human kit (Meso Scale Diagnostics, K15276K).

### RNA-seq

RNA was extracted from ABD and GF-SAT snap frozen tissues of the 17 CTRL and 15 PMOS participants using the gentle MACS dissociator and the RNeasy lipid tissue kit (QIAGEN).

RNA-seq libraries were prepared and sequenced using standard Illumina protocols for a HiSeq 2500 instrument.

### snRNA-seq

Nuclei were isolated from frozen ABD and GF adipose tissue (*n* = 5 CTRL and 5 PMOS; BMI = 30.7 ± 4.39 kg/m^2^, age = 35 ± 5, WHR = 0.85 ± 0.05) as described in ref. 32. Single-nuclei RNA-seq was performed as described in ref. 33. Multiplexed sequencing libraries were generated from cDNA using the Illumina Nextera XT protocol and 150 bp paired-end sequencing was performed on an Novogene SeqXPlus instrument at an average sequencing depth of 400M per library.

### Isolation of stoma vascular cells and scRNA-seq

SVF was isolated from fresh ABD and GF adipose tissue from 5 CTRL and 5 PMOS participants (BMI = 30.7 ± 4.62 kg/m^2^, age = 35 ± 5, WHR = 0.84 ± 0.05) as described in ref. 58. After isolation, cells were stained with DAPI and single-cell suspension was aliquoted into 8 wells of a 384-well source plate (4 wells for ABD and 4 wells for GF). scRNA-seq libraries were generated following the full-length SMART-Seq pro protocol (iCELL8, Takara) as described in ref. 33. Libraries were sequenced with NovaseqX Plus at an average sequencing depth of 600M per library.

### Adipose tissue histology and SVF staining

SAT was fixed in 10% formalin, rinsed with PBS, stored in 70% EtOH, embedded in paraffin within 30 days, and sectioned at 5 μm. Immunofluorescence detection of ROBO1 (mouse monoclonal anti human ROBO1, Invitrogen, MA5-50262) was conducted according to standard protocol of the ALexaFluor 555 Tyramide superboost kit (Invitrogen, B40923). Wheat Germ Agglutinin was used to stain the cellular membranes (Invitrogen, W11261) and Hoescht was used to stain the nuclei. Images were captured using a Leica Stellaris 5 Confocal microscope (Leica Microsystems, Germany). Negative control was done without addition of the primary antibody (Supplemental Figure 6C). SVF cells were plated overnight and then fixed with 4% paraformaldehyde solution. Detection of znf423 (polyclonal anti human znf423, Invitrogen, PA5-66798) and ROBO1 (mouse monoclonal anti human ROBO1, Invitrogen, MA5-50262) was determined by standard immunofluorescence protocol using antimouseIgG 633 (Invitrogen, 21052) and AntiRabbit IgG 546 (Invitrogen, A11035) as secondary antibodies. Negative control was done without addition of the primary antibody (Supplemental Figure 6C). The adipocyte size (μm) was estimated by measuring the major diameter of 200 cells from digital microscopic images using NIS-Elements AR5.02 (Nikon) software.

### In vitro adipogenesis assay

After isolation of SVF, the cells were plated and grown in αMEM/2.5% FBS media complemented with hEGF and hFGF (10 μg/mL and 4 μg/mL, respectively). At confluence the cells were frozen in αMEM/10% DMSO and preserved in LN2. One vial of each subpopulation was thawed to perform the adipogenesis assays. Briefly, cells were plated, grown, and differentiated in DMEM/F12 medium (final concentration of 50 nM insulin, 100 nM dexamethasone, 0.25 mM inhibitor 1-methyl-3-isobutylxanthine, 33 μM biotine, 17 μM D-Panthotenate, 2 nM T3 and 10 μg/mL transferrine and 100 nM rosiglitazone) for 6 days. Next, this medium was replaced by DMEM/F12 (final concentration of 50 nM insulin, 33 μM biotine, 17 μM D-Panthotenate, 2 nM T3 and 10 μg/mL transferrine). The medium was changed every 2 days until 12 days. Cells were harvested before, during and after differentiation (D0, D1, D2, D4, D6, D8, and D12), RNA was extracted, cDNA was generated by reverse transcriptase and target genes were measured by real-time RT-PCR using a ViiA7 sequence detection system (Life technologies) and SyberGreen technology suitable for relative gene expression quantification using the following parameters: one cycle of 95°C for 10 minutes, followed by 40 cycles at 95°C for 15 seconds and 60°C for 1 minute. To test the effect of SLIT2, 2 μg/mL of hSLIT2 recombinant protein (R&D Systems, 9379-SL) was added in the proliferation media at day –1 and in the differentiation media at D0 and D2. Cells were harvest at D4. To test the effect of testosterone (T), T (Sigma-Aldrich, 58-22-0) was added in the differentiation media every other day. Formation of lipid droplets was observed by phase contrast microscopy (Nikon) after staining with Oil red O and quantified by calorimetry.

An additional vial of ADSCs was thawed, seed and culture 24 hours in αMEM/2.5%FBS. The condition media was collected, centrifuge 10 minutes at 12,000*g* and transfer to an ELISA plate to measure SLIT2 level (human SLIT2 ELISA kit, Novus Biologicals, NBP2-82536).

### Bioinformatics

#### sc/snRNA-seq.

Initial analyses of the single cell libraries were performed using CogentAP Analysis Pipeline (Takara Bio), using GRCh19 as a genome reference. Cell and gene filtering and clustering were performed in R package Seurat. Cells were removed is they had < 500 genes and > 15 % of mitochondrial reads for the nuclei and > 30% for the cells. Outlier cells were also filtered if they were in the top and bottom 2.5% percentile for reads and features. Data were normalized in Seurat with SCTransform (59). Each chip was initially clustered separately using 5,000 highly variable genes to detect chip-dependent cell-type clusters. Data were then adjusted for ambient RNA with decontX (60) with cell cluster labels used as the Z parameter. Data were then integrated by using Harmony, each participant was represented in each cell cluster (Supplemental Figure 1A for AT and Supplemental Figure 2A for SVF). Integrated clusters were visualized with UMAP. Differential gene expression analysis was performed using a Wilcoxon rank sum test with Seurat’s “FindMarkers” function. A hypergeometric test was used to assess over-representation of upregulated genes (log_2_ FC > 0.25) from each cluster using the hypeR package and the Hallmark database (61, 62) with genes detected in each cluster used as background. Same function with log_2_ FC > 0.1 was used to determine DEG between groups.

Estimated cell-type proportions were generated using our optimized adipose tissue deconvolution pipeline (37). Briefly, a signature matrix was generated per group and per depot by aggregating counts for each cell type. Each signature matrix was filtered to 6,000 high variable genes. Bulk RNA-Seq counts were converted to CPM and then split by group and depot prior to being deconvoluted with their respective signature matrices using algorithm dtangle.

#### Pseudotime analysis.

To investigate transcriptional dynamics and lineage progression of adipose-derived stromal cells, pseudotime trajectory analysis was performed using the Monocle R package (v2). scRNA-seq data were first processed using Seurat, and relevant cell populations representing stromal progenitors and differentiated states were subset for downstream analysis. Corresponding metadata and gene annotation were constructed and used to initialize a CellDataSet object with a negative binomial expression family. Size factors and dispersions were estimated using Monocle’s internal functions. Genes expressed in at least 10 cells were retained, and an unsupervised selection of ordering genes was performed based on mean expression ≥ 0.1. Dimensionality reduction was carried out using the DDRTree algorithm [reduceDimension()], and cells were ordered along a trajectory with the orderCells() function. Cell trajectories were visualized and colored by state, cell type, condition, and pseudotime. Additional visualizations included density plots and quasirandom scatter plots to depict pseudotime distributions across cell types and experimental conditions.

#### Cell-cell communication analysis.

To investigate intercellular signaling dynamics in adipose tissue, cell-cell communication analysis was performed using the CellChat R package (v2). scRNA-seq data were preprocessed using Seurat, and cells from selected stromal and adipocyte lineages were subset for analysis. Samples were stratified by experimental condition (e.g., CTRL and PMOS) and adipose depot (e.g., ABD), and CellChat objects were generated for each group. The CellChatDB.human database was used, focusing on nonprotein signaling pathways in adipose tissue and extracellular matrix receptor pathways in SVFs. CellChat’s internal functions were used to preprocess and filter the data, including identifyOverExpressedGenes(), identifyOverExpressedInteractions(), and projectData() using the PPI.human network. Communication probabilities and pathway-level interactions were computed using computeCommunProb() and computeCommunProbPathway(), followed by aggregation with aggregateNet(). Merged analyses using mergeCellChat() enabled comparative exploration of signaling differences between CTRL and PMOS samples. Centrality scores were calculated to evaluate the roles of different cell types as signal senders and receivers. Visualization of signaling dynamics was performed using chord diagrams and heatmaps [netVisual_aggregate(), netVisual_chord_gene()]. Differential signaling was assessed using presto-based differential expression testing through identifyOverExpressedGenes(), followed by ligand-receptor filtering via netMappingDEG() and subsetCommunication(). Comparative analysis focused on upregulated and downregulated hormone-related signaling (e.g., testosterone, DHT, cholesterol) between groups.

#### Bulk RNA-seq.

Raw reads were aligned to the hg19 genome with STAR using nf-core/RNA-seq pipeline version 3.2. Genes counts were quantified with Salmon using default parameters. The differentially expressed genes analysis between ABD and GF was performed using edgeR and DESeq2, with the standard RNA-seq workflow; genes with counts < 50 were filtered.

#### WGCNA.

Gene expression data were collected from adipose tissue (ABD and GF depots) of female participants and normalized as counts per million (CPM). Clinical phenotypes included free testosterone, total fat mass, VAT mass, leg fat percentage (LFM %), liver fat percentage (Liver Fat %), and intramuscular adipose tissue volume (IMAT). Genes and samples with excessive missing values were removed using the goodSamplesGenes() function from the WGCNA package (version 1.69) in R. After quality control, hierarchical clustering with average linkage was performed to identify and optionally exclude outlier samples. Clinical traits were matched to the expression data by sample ID and visualized along the sample dendrogram to assess associations. A soft-thresholding power was chosen using the pickSoftThreshold() function to approximate scale-free topology; a power of 6 was selected. Coexpression modules were then identified using the blockwiseModules() function with the following parameters: unsigned topological overlap matrix (TOM), minimum module size of 100, and merge cut height of 0.25. Modules were assigned distinct colors, and their eigengenes (MEs) were computed for correlation with traits. Module-trait relationships were assessed using Pearson correlation between MEs and each clinical trait. Significant associations were visualized in a labeled heatmap. Genes were ranked within each module based on their GS and MM values, and a comprehensive annotation table was produced, including module color, trait significance, and statistical significance (*P* values). Highlighted modules were used for pathway enrichment analysis using HypeR (pathways with –log_10_FDR > 1.3 are represented).

### Statistics

Pearsons correlation between phenotypical traits and adipocyte size, nuclei proportions, or sex hormones were conducted with R Package Hmisc (V5.2-2) rcorr() function. A *P* value less than 0.05 was considered significant. For the clinical data, comparison between groups was made with unpaired 2-tailed *t* test. For in vitro assays, Mann-Whitney (group comparison) or Wilcoxon matched-pairs signed rank (testosterone or SLIT2 effect) test were used.

### Study approval

All procedures were performed under a research protocol approved by the AdventHealth Institutional Review Board.

### Data availability

sc/snRNA-Seq data sets have been deposited at GEO under the accession nos. GSE209618, GSE322696, and GSE322699 and are publicly available as of the date of publication. The paper does not report original code. Values for all data points in graphs are reported in the Supporting Data Values file. Any additional information required to reanalyze the data reported in this paper is available from the lead contact upon request. This study has been registered at ClinicalTrials.gov (NCT04034706).

## Author contributions

AD contributed conceptualization, methodology, investigation, formal analysis, visualization, writing-original draft. EE contributed formal analysis, software, visualization, writing-review and editing. KLW contributed software, formal analysis, review and editing. TFO contributed conceptualization, supervision, funding acquisition, writing- review and editing. SRS contributed conceptualization, supervision, funding acquisition, writing- review and editing.

## Conflict of interest

The authors have declared that no conflict of interest exists.

## Supplementary Material

Supplemental data

Supplemental data set 1

Supporting data values

## Figures and Tables

**Table 1:**
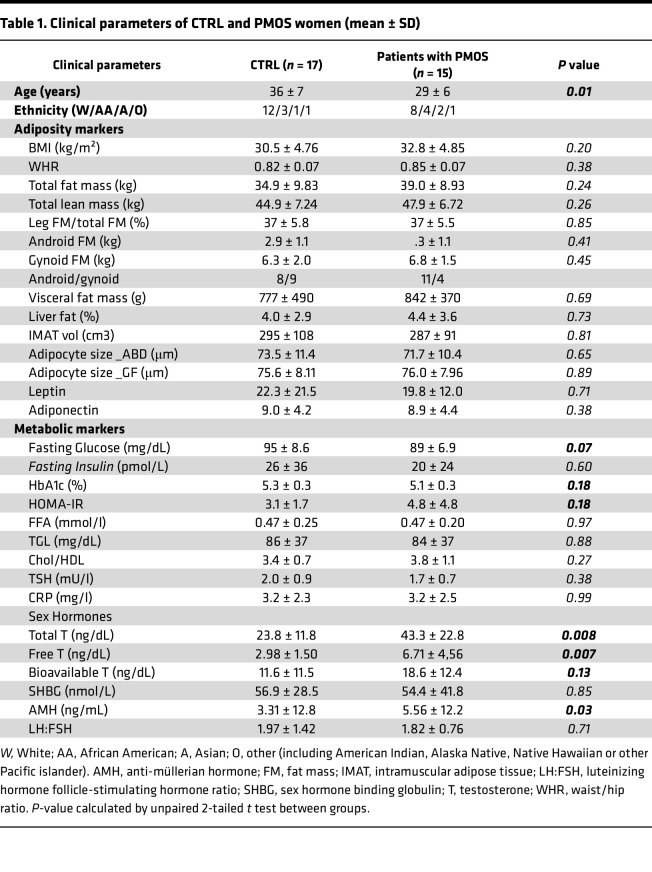
Clinical parameters of CTRL and PMOS women (mean ± SD)

## Version 1 08/10/2026

Electronic publication
