# Supplementary material for: Impaired adipogenesis and lipid storage capacity in subcutaneous adipose tissue of patients with PMOS
Source: JCI Insight. 2026 Aug 10;11(15):e199981. doi: 10.1172/jci.insight.199981 (PMC13463609; doi:10.1172/jci.insight.199981)
Supplement: Supplementary file 1 [file jciinsight-11-199981-s176.pdf]

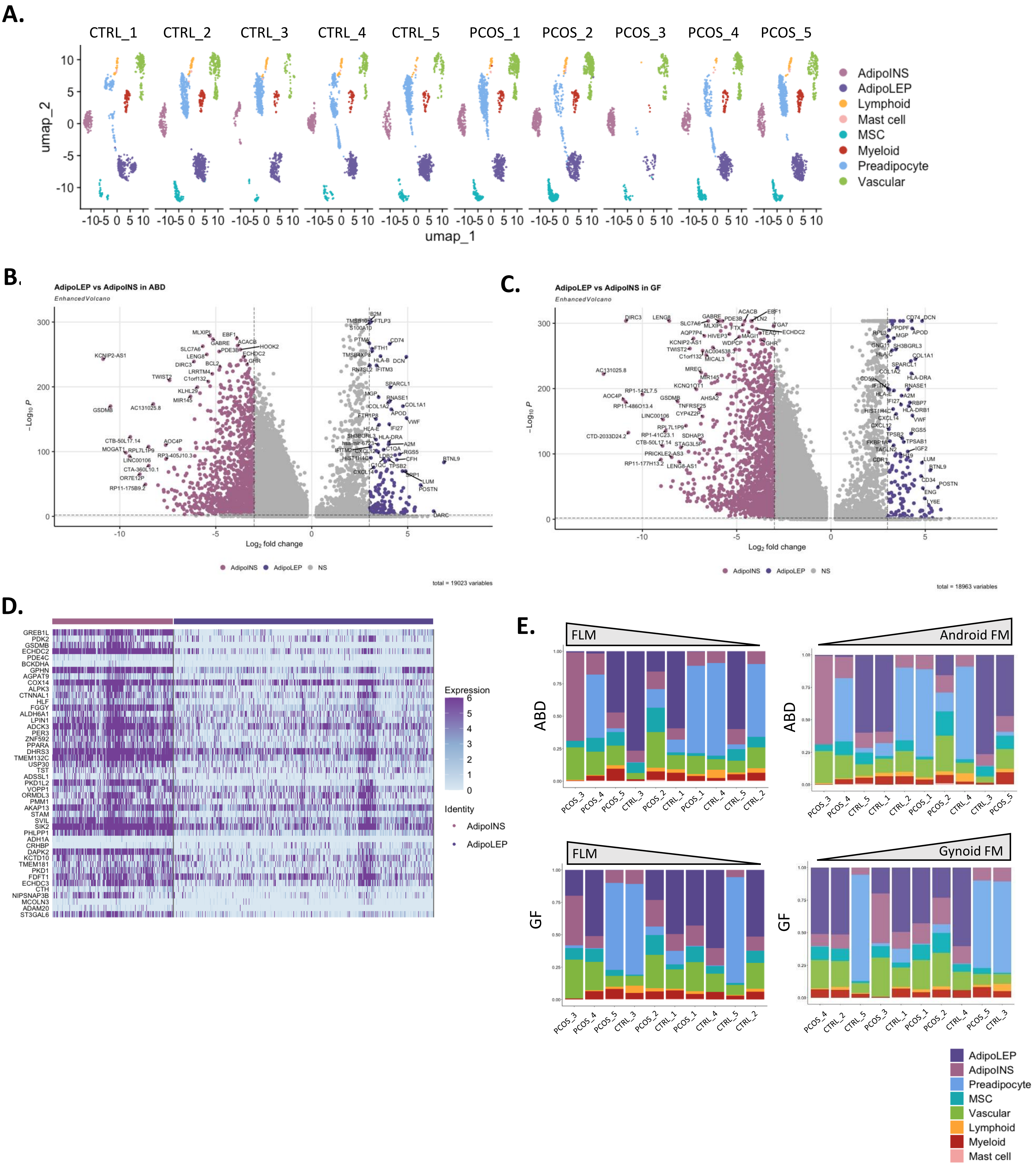

Supplemental Figure S1: (A) UMAPs representation of the snRNA-seq data for each individual participant. (B) Volcano plot showing the differentially expressed genes between AdipoLEP and AdipoINS subpopulations in ABD depot. (C) Volcano plot showing the differentially expressed genes between AdipoLEP and AdipoINS subpopulations in GF depot. (D) Heatmap representation of expression level of genes associated with hyperplasia adipose tissue expansion (defined in Gao *et al*) in AdipoINS nuclei and in AdipoLEP nuclei in ABD depot. (E) Stacked plots showing the relative proportion of each cell subpopulations according to the fat leg mass (FLM), android fat mass (FM) and gynoid FM.

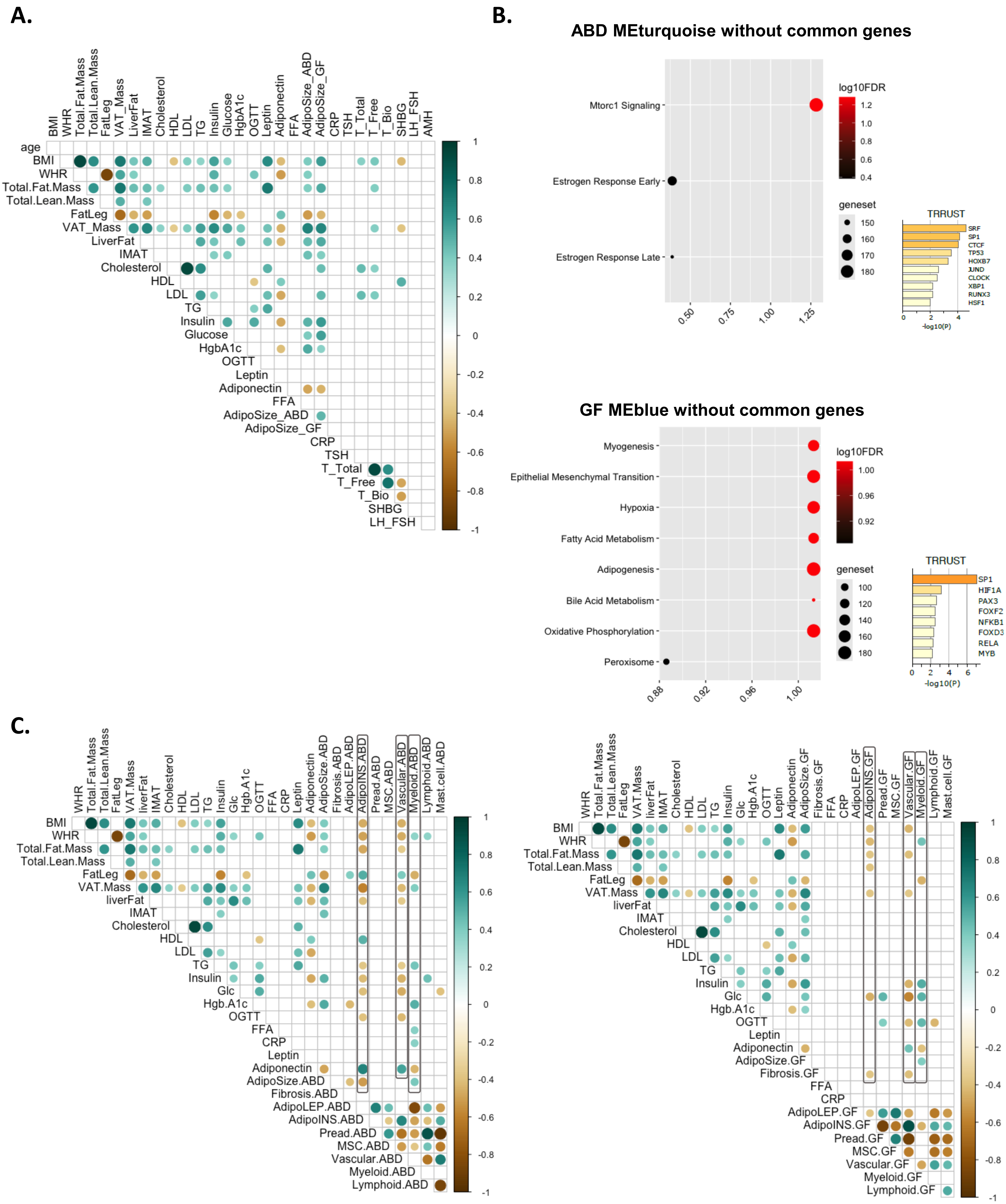

Supplemental Figure S2: (A) Correlation matrix of relevant clinical parameters and sex hormones levels in the 32 subjects (B) Functional enrichment analysis performed on the unique genes that belong to the turquoise module in ABD-AT and blue module in GF-AT. TRRUST database was used to specifically identified transcriptional regulator. (C). Correlation matrix of relevant clinical parameters and cell percentage estimated by deconvolution analysis in ABD-SAT (left) and GF-SAT (right). N=15 PCOS and 17 control.

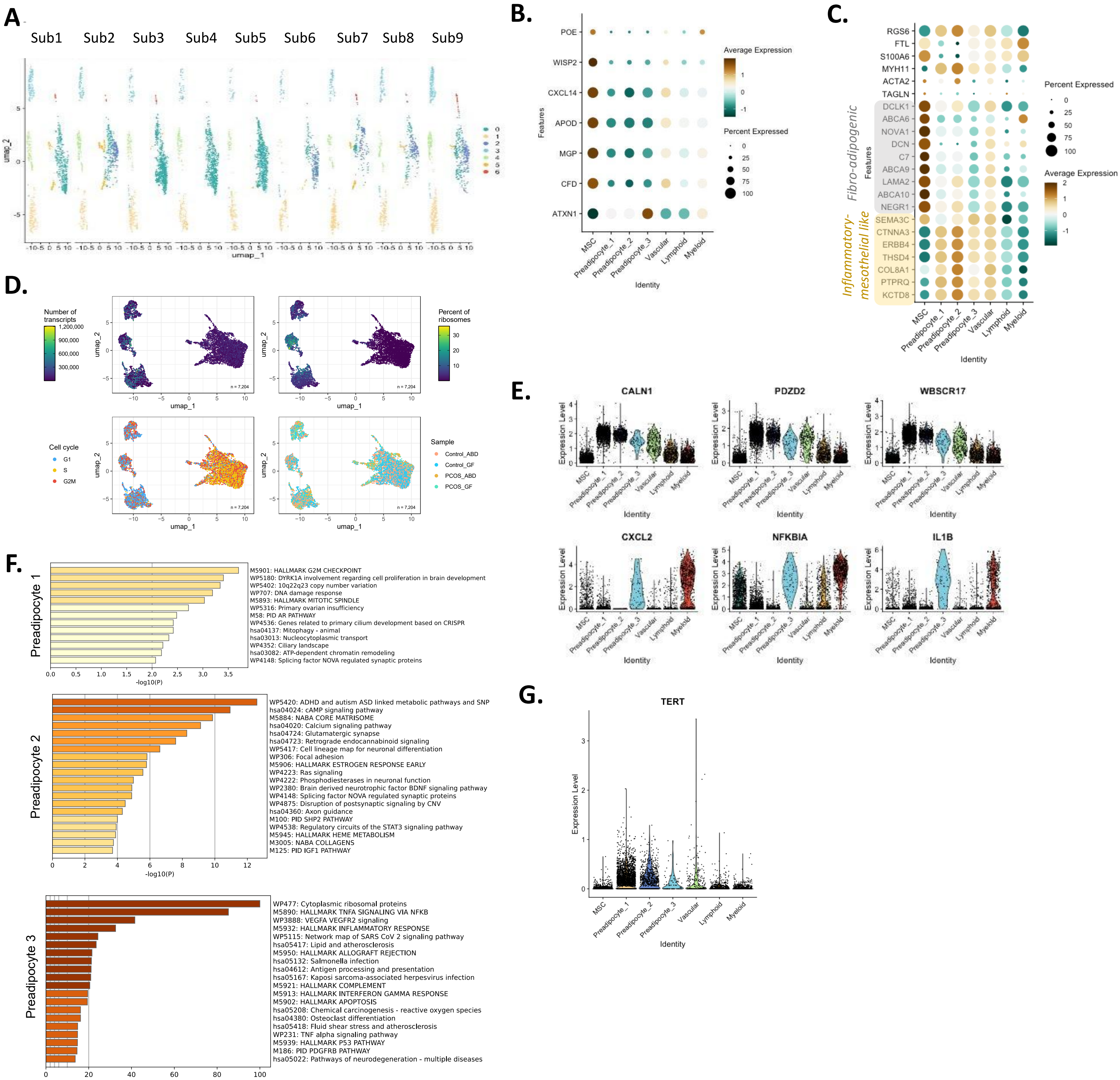

Supplemental Figure S3: (A) UMAPs of the scRNA-seq data for each individual participant. (B) Dotplot representation of relative expression of marker genes used to identify each cluster in the SVF-scRNA-seq dataset. (C) Dotplot representation of relative expression of genes identified by Strieder Barboza *et al* in each cell clusters. (D) Feature plots showing the number of transcripts, the percent of ribosome, the cell cycle phase and the grouping (ABD vs GF and CTRL vs PCOS) of each sample. (E) Violin plots comparing expression level of preadipocyte 1 and 2 marker genes (top) and preadipocyte 3 marker genes (bottom). (F) Bar plots showing pathways enriched in each preadipocyte populations (preadipocyte compared to all other cell types) . (G) Violin plot showing expression of *TERT* gene in each cell cluster.

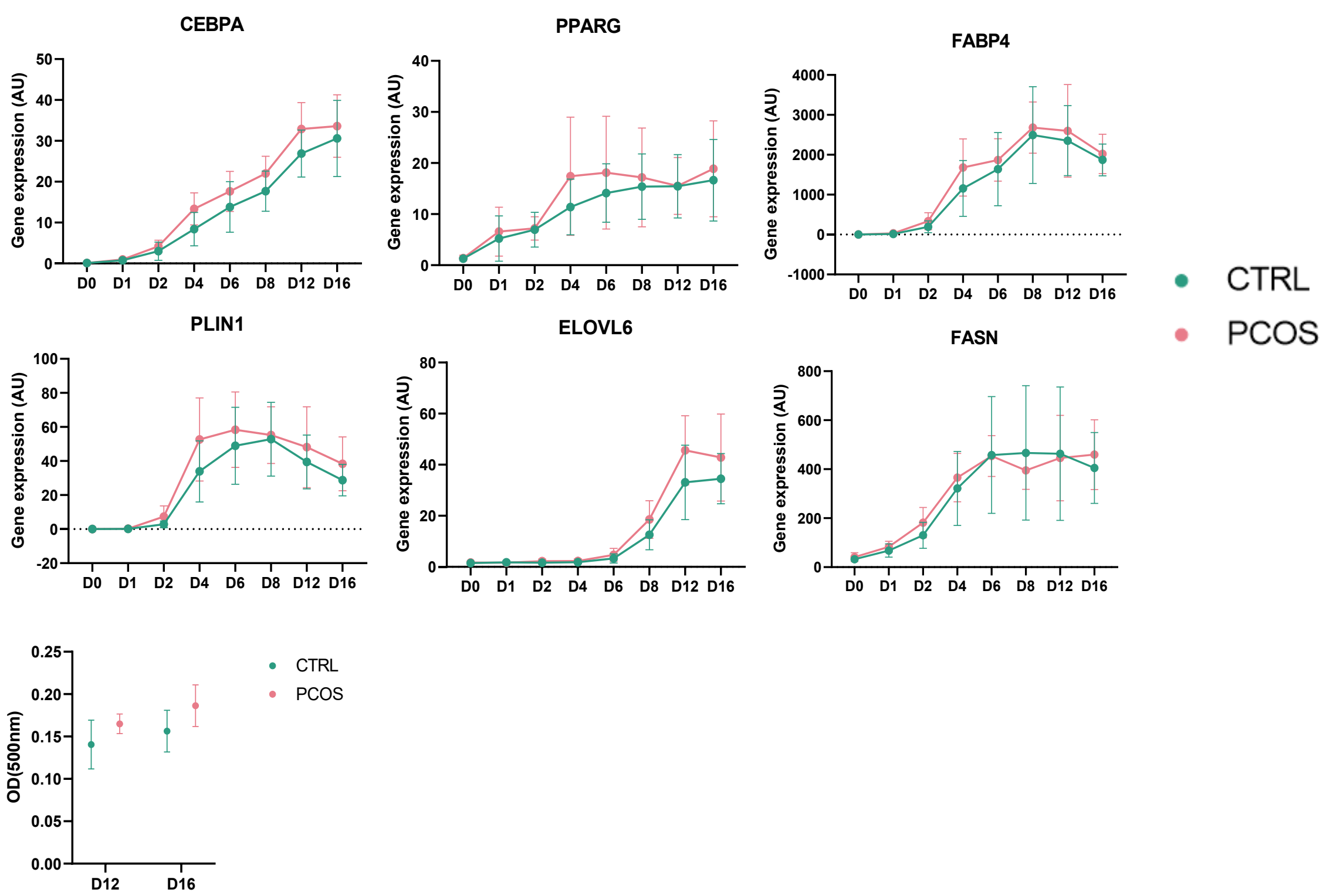

Supplemental Figure S4: Gene expression level during 12-days of adipogenic differentiation of GF-MSC isolated from CTRL-SVF (n=5) and PCOS-SVF (n=5). The degree of differentiation was measured by Oil Red O in each group after 12 days (D12) and 16 days (D16) of differentiation.

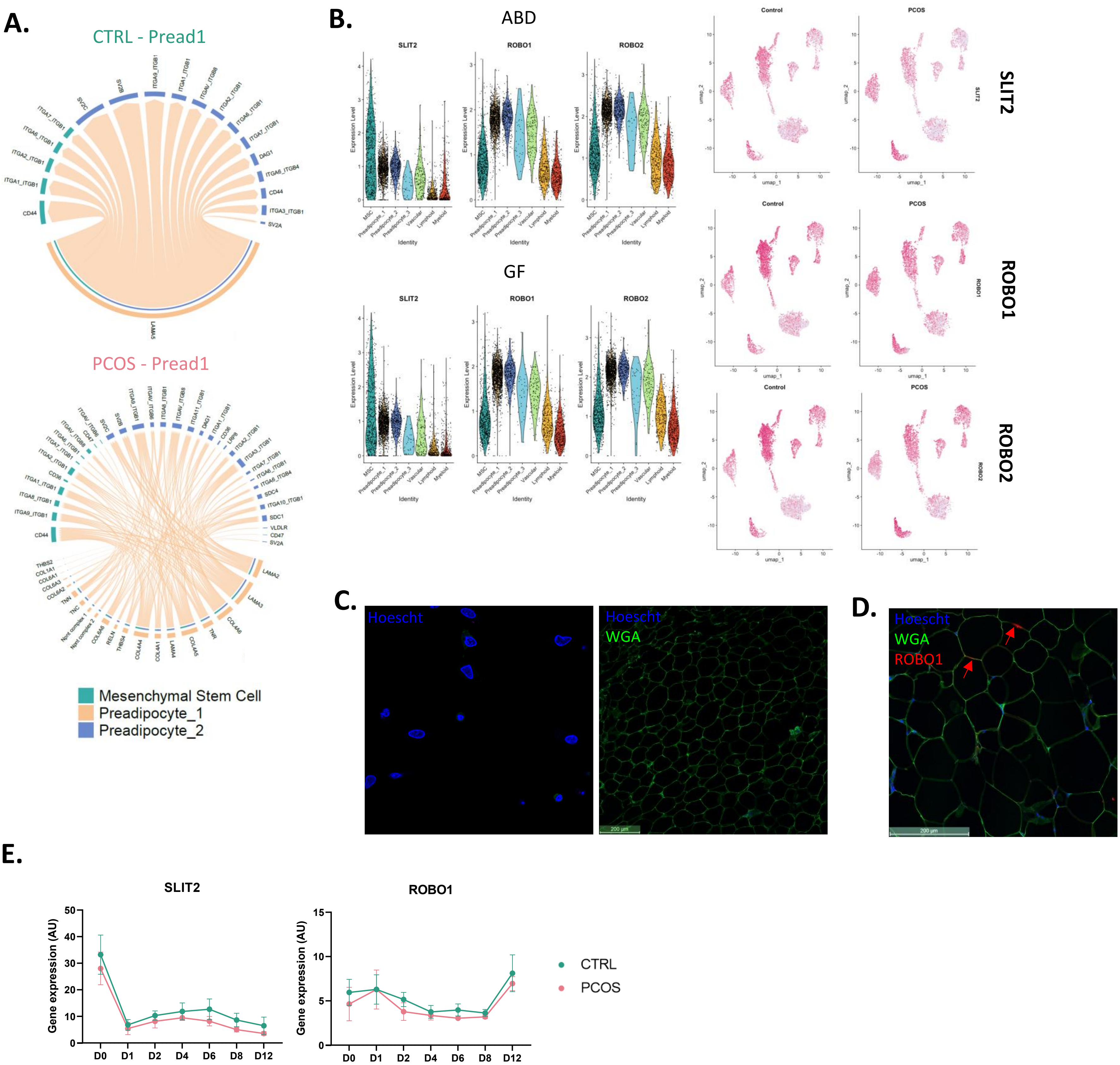

Supplemental Figure S5: (A) Circle plots of secreted signaling pathways coming from Preadipocyte 1 population enriched in CTRL and in PCOS group. The colors of the circle specify which cell type receives the signal. (B) Violin plots comparing the expression level of SLIT2, ROBO1 and ROBO2 in each clusters in ABD and GF depot. Feature plots showing expression of SLIT2, ROBO1 and ROBO2 in CTRL and PCOS group. (C) Negative control of immunofluorescence staining of stroma vascular fraction cells and subcutaneous adipose tissue using only 2nd antibodies and hoescht (for SVF) and WGA (for AT). (D) Immunofluorescence staining of ABD subcutaneous adipose tissue staining using WGA (488) and ROBO1 (647) antibodies. Hoescht was used to stain the nuclei. (E) Immunofluorescence staining of using ZNF423 (488) and ROBO1 (647) antibodies. Hoescht was used to stain the nuclei. (E) *SLIT2* and *ROBO1* expression level during 12-days of adipogenic differentiation of ABD-MSC isolated from CTRL-SVF (n=5) and PCOS-SVF (n=5).

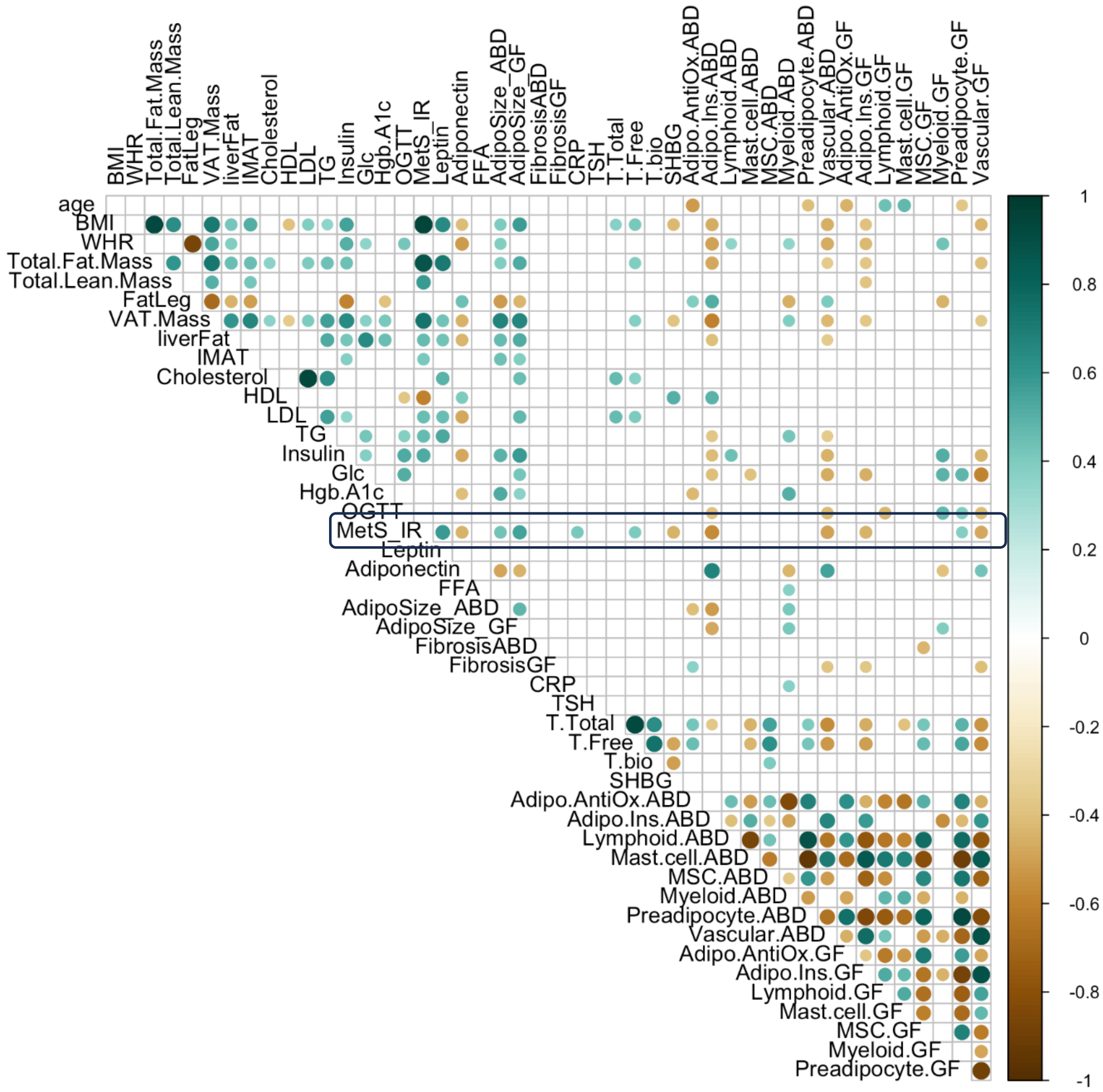

Supplemental Figure S6: Correlation matrix of relevant clinical parameters including the METS-IR score and cell percentage estimated by deconvolution analysis in ABD-SAT and GF-SAT in the 32 subjects.
